# Telomeric *Trans-*Silencing in *Drosophila melanogaster*: Tissue Specificity, Development and Functional Interactions between Non-Homologous Telomeres

**DOI:** 10.1371/journal.pone.0003249

**Published:** 2008-09-22

**Authors:** Thibaut Josse, Corinne Maurel-Zaffran, Augustin de Vanssay, Laure Teysset, Anne-Laure Todeschini, Valerie Delmarre, Nicole Chaminade, Dominique Anxolabéhère, Stéphane Ronsseray

**Affiliations:** 1 Laboratoire “Dynamique du Génome et Evolution”, Institut Jacques Monod, UMR7592, CNRS-Universités Paris 6 et 7, Paris, France; 2 Laboratoire “Biologie du Développement”, UMR7622, CNRS-Université Paris 6, Paris, France; 3 Laboratoire “Evolution, Génomes, Spéciation”, CNRS UPR 9034, Bât 13, Gif sur Yvette, France; Baylor College of Medicine, United States of America

## Abstract

**Background:**

The study of *P* element repression in *Drosophila melanogaster* led to the discovery of the telomeric *Trans*-Silencing Effect (TSE), a homology-dependent repression mechanism by which a *P*-transgene inserted in subtelomeric heterochromatin (Telomeric Associated Sequences, “TAS”) has the capacity to repress in *trans*, in the female germline, a homologous *P-lacZ* transgene located in euchromatin. TSE can show variegation in ovaries, displays a maternal effect as well as an epigenetic transmission through meiosis and involves heterochromatin and RNA silencing pathways.

**Principal Findings:**

Here, we analyze phenotypic and genetic properties of TSE. We report that TSE does not occur in the soma at the adult stage, but appears restricted to the female germline. It is detectable during development at the third instar larvae where it presents the same tissue specificity and maternal effect as in adults. Transgenes located in TAS at the telomeres of the main chromosomes can be silencers which in each case show the maternal effect. Silencers located at non-homologous telomeres functionally interact since they stimulate each other *via* the maternally-transmitted component. All germinally-expressed euchromatic transgenes tested, located on all major chromosomes, were found to be repressed by a telomeric silencer: thus we detected no TSE escaper. The presence of the euchromatic target transgene is not necessary to establish the maternal inheritance of TSE, responsible for its epigenetic behavior. A single telomeric silencer locus can simultaneously repress two *P-lacZ* targets located on different chromosomal arms.

**Conclusions and Significance:**

Therefore TSE appears to be a widespread phenomenon which can involve different telomeres and work across the genome. It can explain the P cytotype establishment by telomeric *P* elements in natural *Drosophila* populations.

## Introduction

Mobilization of transposable elements (TEs) is regulated by complex mechanisms which involve proteins encoded by the TEs themselves as well as heterochromatin formation and small RNA silencing mechanisms [1]–[8]. The study of these mechanisms can be facilitated by the fact that some of these elements are recent components of genomes, allowing us to study strains with and without these transposable elements (TEs). The *P* transposable element (a transposase encoding TE) has invaded all natural populations of *Drosophila melanogaster* in less than two decades during the last century (1950–1970) [9], [10]. Strains free of *P* elements (collected before 1950) are called M strains, whereas strains with *P* elements (collected after 1970) are called P strains [11], [12]. P strains crossed with each other do not show *P* elements mobilization. However, when P males are crossed to M females, *P* elements repression is lifted in the germline of the resulting progeny. This induces the occurrence of a syndrome of germline abnormalities called hybrid dysgenesis (P-M system) which includes a high mutation rate, chromosomal rearrangements, male recombination and an agametic temperature-sensitive sterility called GD sterility (Gonadal Dysgenesis) [11]–[13]. *P* element mobility and dysgenesis can be repressed by various mechanisms depending on the structure and location of regulatory *P* copies [14], [15]. A biparentally-transmitted moderate repression can be established by different types of *P* copies having the capacity to encode polypeptides (deleted transposase) which behave as repressors [16]–[21]. In contrast, the most efficient *P* repression mechanism is a maternally transmitted *P* repression capacity termed “P cytotype” [14], [22] - the absence of *P* repression, being referred to as “M cytotype”. P cytotype determination appears to involve mainly a master locus located at the telomere of the *X* chromosome at the cytological site 1A [23]–[26]. Indeed, one or two complete or defective *P* elements at 1A repress *P* transposition and *P*-induced hybrid dysgenesis more efficiently than 15–20 *P* elements scattered at random on the chromosomes, following *P* element transformation of an M line [23], [25]–[29]. Further, establishment of the P cytotype by telomeric *P* insertions was shown to be sensitive to mutants affecting both heterochromatin formation (HETEROCHROMATIN PROTEIN 1, “HP1”) and small RNA silencing pathways (AUBERGINE, an Argonaute member), suggesting a complex molecular mechanism [25], [30], [31]. These telomeric *P* elements at 1A were found to be inserted in a sub-telomeric heterochromatin region [25], [26], [32] called “Telomeric Associated Sequences” (TAS) [33], [34]. TAS are heterochromatic tandemly-repeated non-coding sequences that induce variegation in the eye of *P-white* transgenes inserted within them [34]–[41]. Recently, extensive analysis of small RNAs complexed with Piwi family proteins (AUBERGINE, PIWI and AGO3) were performed in the *Drosophila* female germline [6], [42]. This analysis showed that most of these piwi-RNAs (piRNAs) correspond to repeat associated small interfering RNA (rasiRNA [3], [7]). Among them, piRNAs corresponding to TAS regions were found, suggesting that TAS may correspond to platforms of piRNA production [6], [43].

Finally, not only telomeric natural *P* elements, but also telomeric *P-* transgenes, which are unable to encode any *P*-repressor, were shown to have some repressive capacities. Indeed, a *P-lacZ* transgene located in TAS can repress an euchromatic *P-lacZ* transgene in *trans*, a phenomenon termed “*Trans*-Silencing Effect” (TSE) [44]. This repression is dependent on the length of homology between the two sequences [26]. Incomplete TSE does not result in homogenous weak lacZ staining but produces clear-cut variegation of *lacZ* expression in the germline [45], [46]. TSE appears to illustrate the molecular mechanism of the strong *P* repression elicited by telomeric *P* elements since P cytotype and TSE present similar properties: 1- both show maternal inheritance and epigenetic transmission through the meiosis which depends on an extra-chromosomal maternally-transmitted factor [45], [47]; 2- both are sensitive to mutations in *Su(var)205* encoding HP1 [25], [45]; 3- both are sensitive to mutations of *aubergine* affecting RNA silencing [31], [45]. Recently, we conducted a more extensive candidate gene analysis of mutations affecting TSE and have shown that this silencing strongly depends on genes involved in the rasiRNA silencing pathway (*aubergine, homeless, armitage* and *piwi*), but does not depend on *r2d2* involved in the small interfering RNA (siRNA) silencing pathway, nor on *loquacious* involved in the microRNA (miRNA) pathway [45]. These data support the proposition that TSE involves a rasiRNA pathway linked to heterochromatin formation which was co-opted by the *P* element to establish repression of its own transposition after its recent invasion of the *D. melanogaster* genome.

In this paper, we analyze the phenotypic properties of TSE, first showing that this silencing is restricted to the female germline and second that it occurs during development in third instar larvae. We further investigate its generality throughout the genome: we identify new telomeric silencers located in the TAS of the second and third chromosome telomeres and show that silencers located at non-homologous telomeres can functionally interact in establishing TSE. We show that TSE represses all tested euchromatic insertions located on all main chromosomal arms. We finally also show that TSE can repress simultaneously two transgenes located on different chromosomal arms. Therefore, TSE properties allow us to propose that the TAS located at non-homologous telomeres can be interacting piRNAs-producing platforms. Consequently, regulatory *P* element copies can be inserted in these platforms and interact to establish repression of the euchromatic *P* copies scattered throughout the genome.

## Materials and Methods

### Experimental conditions

All crosses were performed at 25°C and involved 3–5 couples in most of the cases. All ovary *lacZ* expression assays were carried out using X-gal overnight staining as described in Lemaitre *et al.* 1993 [48], except that ovaries were fixed for 6 min [45].

### Transgenes and strains

#### Transgene structures

*P-lacZ* fusion enhancer-trap transgenes *(P-1152*, *P-1103, P-1155, BQ16*, *BC69, BA37, P-1039*) contain an in-frame translational fusion of the *E. coli lacZ* gene to the second exon of the *P transposase* gene and contain *rosy* ^+^ as a transformation marker [49]. *SUPor-P-863-1, P-w-y-T2R-PAR* are *P-yellow-white* transgenes [50] (see legend of Figure S1). *P-Co1* is an insertion of the *pCo* transgene (*P-otu-lacZ*) in which β−galactosidase expression is driven by the *otu* promoter and is therefore strongly detected in both nurse cells and the mature oocyte [51]. This transgene contains a *white* gene as a transformation marker. *A4-4* (also called *P-833*) is a *P-white-rosy* transgene [34], [52], [53].

#### Telomeric silencers

The *P-1152* and *P-1103* insertions come from stocks #11152 and #11103 of the Bloomington Stock Center and have been mapped at the telomere of the *X* chromosome (cytological site 1A); these stocks were previously described to carry a single *P-lacZ* transgene inserted in TAS [44]. However, in our #11152 stock, we have mapped two *P-lacZ* transgenes inserted in the same TAS unit and in the same orientation which might have resulted from an unequal recombination event duplicating the *P-lacZ* transgene [45]. *P-1155* comes from stock #11155 of the Bloomington stock center. It contains a single *P-lacZ* transgene in TAS at the *3R* chromosome arm telomere (site 100F). *P-1152* and *P-1103* show no *lacZ* expression in the ovary, whereas *P-1155* shows weak and non-uniform lacZ staining in follicle cells but no staining in the germline (data not shown). *SUPor-P-863-1* has been mapped to TAS of the *X*-chromosome telomere [50] and carries two adjacent *SUPor-P* transgenes (*P-white-yellow* [50]) in the same orientation, one of which is deleted at one extremity (see legend of Figure S1). *P-w-y-T2R-PAR* has been mapped to TAS of the *2R* chromosomal arm telomere (site 60F) and carries a single *P-white-yellow* transgene (see legend of Figure S1). *A4-4* has been mapped to TAS of the *3R* chromosomal arm telomere (site 100F) and carries a single *P-white-rosy* transgene [34], [43], [52], [53]. All these telomeric silencers are homozygous viable and fertile. Information concerning the mapping of telomeric silencers within a TAS repeat are shown on Figure S1.

#### Euchromatic targets

*BC69* is inserted on chromosome *2* in the first exon of the *vasa* gene and results in *vasa* loss of function: it is consequently homozygous female sterile. *BQ16* is located at 64C in euchromatin of the third chromosome and is homozygous viable and fertile. It was mistakenly reported to be located on the second chromosome in [46]. *BQ16* and *BC69* are strongly expressed in the nurse cells and in the oocyte. *BA37* is located at 87F on the third chromosome and is homozygous lethal. It is strongly expressed in the follicle cells but shows no expression in the female germline. *P-1039* is located at 60B on the second chromosome and is homozygous lethal. It shows strong lacZ staining in numerous tissues including the follicle cells, the nurse cells and the oocyte. *BA37* and *P-1039* are maintained over balancer chromosomes. *P-Co1* is an insertion of the *pCo* transgene (*P-otu-lacZ*) on the third chromosome (87AB) which is homozygous viable and fertile. *ptc-lacZ* and *sd-lacZ* correspond to enhancer-traps in the *patched* and *scalloped* genes, respectively. Other constructs which have been used as TSE targets are listed in **Table S1**. **Table S2** gives the nomenclature information and references for all transgenes tested as TSE silencers or targets.

Lines carrying transgenes have M genetic backgrounds (devoid of *P* transposable elements), as well as the multi-marked balancer stocks used in genetic experiments (*M5; Cy/T(2;3)ap^Xa^* and *M5; TM3, Sb/T(2;3)ap^Xa^*). Canton^y^ and *w* ^1118^ are typical M strains marked with a spontaneous mutation of *yellow* and a deletion inside the *white* locus, respectively.

### Quantification of TSE

Depending on the target, TSE can be almost total or intermediate. When TSE is incomplete, variegation is observed since “on” and “off” *lacZ* expression is seen among egg chambers: egg chambers can show strong expression (dark blue) or no expression, but intermediate repression levels are not (or very rarely) found. Simple quantification of TSE is thus possible by determining the percentage of repressed egg chambers. The number of repressed chambers among the first five egg chambers of a given ovariole is scored for ten ovarioles chosen at random per ovary. For a given genotype more than 1000 egg chambers were classically counted. This measure generally produces very reproducible results among replicate experiments allowing accurate quantification of TSE [45].

## Results

### TSE is germline specific

**Figure 1:**
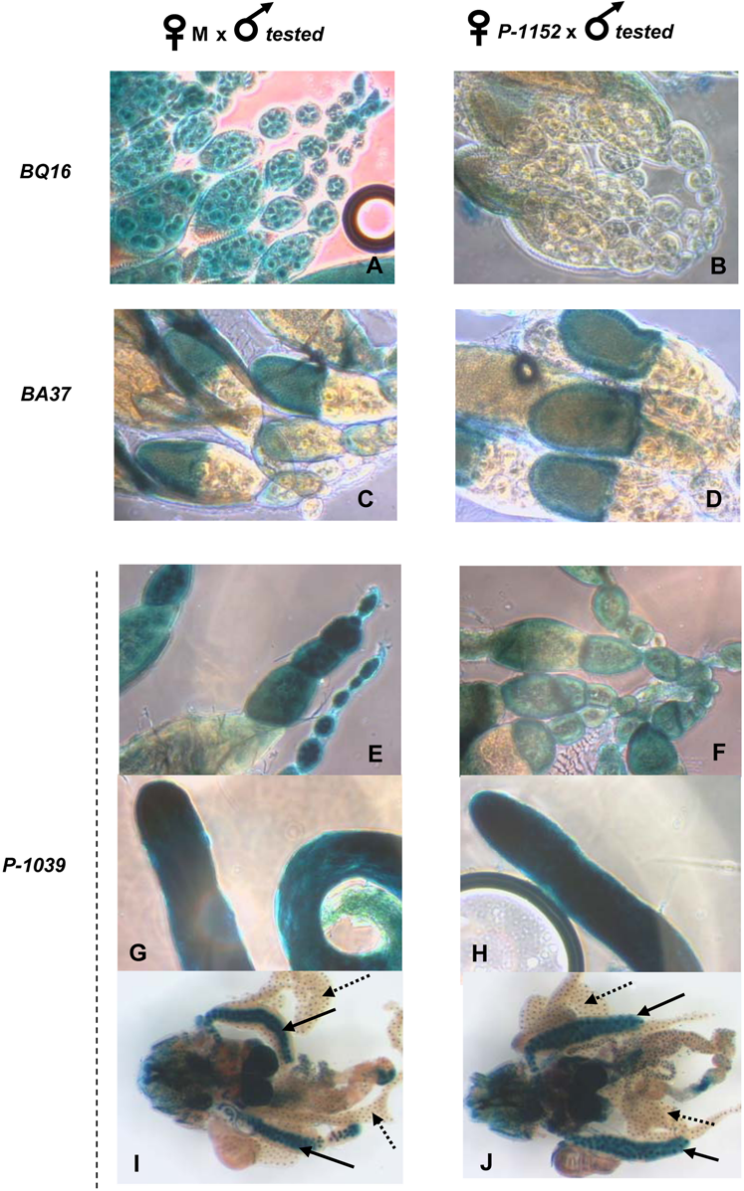
TSE is restricted to the female germline. Males from lines carrying various tested euchromatic *P-lacZ* enhancer-trap transgenes expressed in different tissues were crossed with females devoid of *P*-transgenes (M females) or with females carrying the telomeric silencer *P-1152*. G_1_ individuals were stained overnight for lacZ activity. A–F, adult ovaries; G–H, adult testis; I–J, third instar larvae salivary glands (full arrow) and fat body (dashed arrow). The staining observed in the larval brain is not discussed in the present analysis since the *P-1152* transgene alone produces staining in this tissue. The enhancer-traps tested as targets and introduced by the fathers are indicated on the figure (on the left) and their structures and locations are described in the “Material and Methods”.

*Trans*-silencing was discovered through the study of the mechanism of the establishment of the P cytotype, the *P* element maternally-inherited repressive state which takes place in the germline, the tissue in which the *P* elements can transpose and induce hybrid dysgenesis. It was shown previously that a crucial component of this P cytotype results from telomeric *P* elements inserted in subtelomeric heterochromatin at the telomere of the *X* chromosome [25]. These telomeric *P* elements have repressive capacities which are restricted to the germline. We have thus tested the tissue specificity of TSE in order to determine if it can take place in other tissues than the germline. Males which carry euchromatic *P-lacZ* transgenes expressed in various tissues were crossed with M females and with females carrying a strong telomeric silencer locus (*P-1152*) [45]. Various tissues in G_1_ individuals were stained and were compared for the two kinds of G_1_ progeny. **Figure 1** shows that, for the targets expressed in ovaries, TSE occurs in the female germline (nurse cells inside egg chambers, see *BQ16* (A *vs* B) and *P-1039* (E *vs* F)) but does not occur in the somatic follicle cells surrounding the egg chambers (see *BA37* (C *vs* D) and *P-1039* (E *vs* F)). Further, no repression by *P-1152* is detected in the testis (see *P*-*1039*, G *vs* H). Finally, no TSE was detected in the salivary glands nor in the fat body of third instar larvae (see *P-1039*, I *vs* J). It can be noted that a given *P-lacZ* insertion (*P-1039*) expressed in various tissues, undergoes repression in the nurse cells but not in the somatic cells nor in the testis. Thus, this tissue specificity cannot be interpreted as a consequence of a specific property of the target genomic site. In addition, the germline repression using the *BQ16* and *P-1039* targets corresponds to TSE since in both cases it was shown to present the TSE signatures *i.e.* a maternal effect (repression only when the telomeric transgene is maternally inherited) and variegation when the repression is not complete ( [45] and data not shown).

To generalize these results, TSE was tested using a number of different *P-lacZ* target insertions scattered through the genome. Nineteen insertions, located on all main chromosomes and showing expression in the female germline (nurse cells and in some cases the oocyte), were tested (Table S1). In all cases, TSE was observed in this tissue. TSE appears to be a general phenomenon which can take place across the whole genome. Further, the tissue specificity of TSE at the adult stage was confirmed with seven different *P-lacZ* target insertions expressed in ovarian somatic follicle cells and with six different *P-lacZ* target insertions expressed in the testis: in all cases no repression was observed. Among these targets, three *P-lacZ* are expressed in both the female germline and in follicle cells and six *P-lacZ* targets are expressed in both the female germline and the testis: in each case TSE was observed only in the nurse cells and/or the oocyte. TSE at the adult stage appears therefore restricted to the female germline and shows no escapers among target transgenes expressed in this tissue, as tested using 19 targets located on all the main chromosomes.

### TSE is also active in larvae

**Figure 2:**
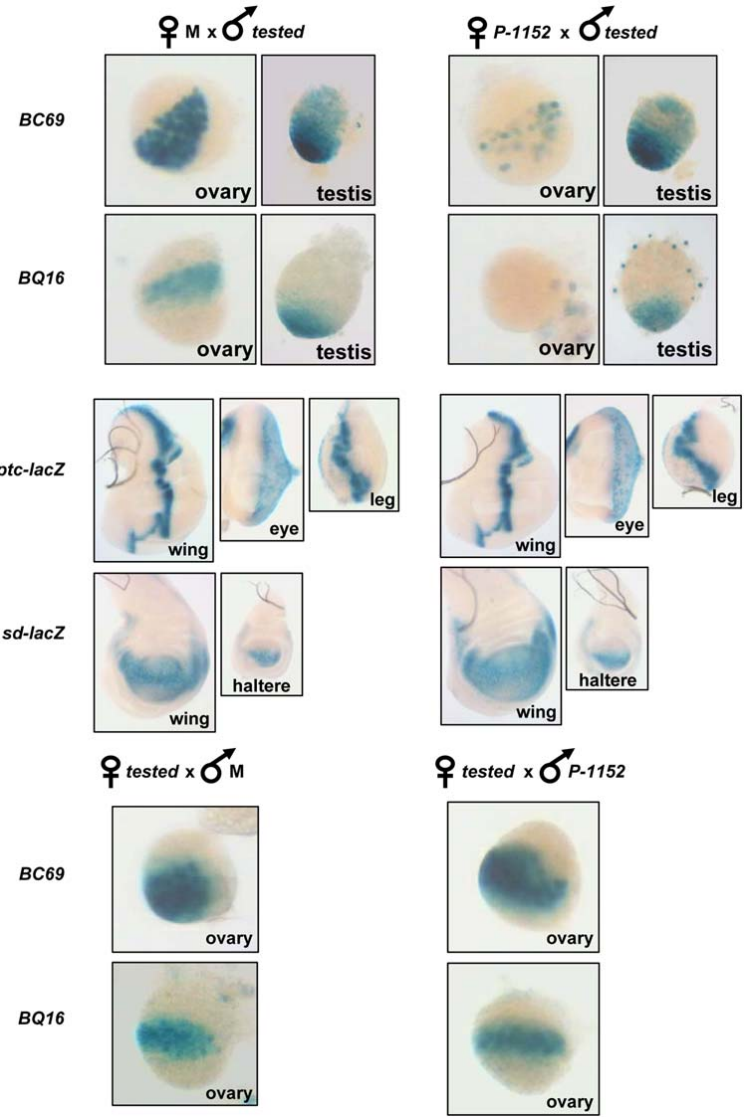
TSE occurs in third instar larvae and presents the same properties as in adults. Different crosses were performed between individuals from M or *P-1152* lines and individuals from lines carrying various euchromatic *P-lacZ* enhancer-trap transgenes expressed in different tissues. Imaginal discs or gonads from G_1_ third instar larvae were stained overnight for lacZ activity. The tissue is indicated on the figure together with the cross (above) and the enhancer-trap used (on the left). *ptc-lacZ* and *sd-lacZ* are enhancer trap in the *patched and scalloped* genes, respectively. *BC69* and *BQ16* are expressed in the germline of the two sexes and are described in the “Material and Methods”. Pictures are not to scale.

TSE was previously analyzed mainly in adults. We tested if TSE can occur during development at the third instar larvae in gonads and imaginal discs **(** **Figure 2** **).** We first performed classical crosses known to induce a strong TSE in adult ovaries. We crossed *BQ16* and *BC69* males with both M females and females carrying the telomeric *P-1152* silencer and have stained the gonads of G_1_ third instar larvae. In female larval gonads, *BQ16* and *BC69* were expressed in the G_1_ deriving from M females but were strongly repressed in G_1_ larvae deriving from *P-1152* females (Figure 2). Conversely, no repression by *P-1152* was observed in the male gonads for the two target transgenes. Therefore, TSE is detected in female but not male gonad tissue in larvae. We further tested the maternal effect of TSE by performing the reciprocal cross (females *P-lacZ* target x males *P-1152*) and no repression was detected in G_1_ female larvae. Finally, we tested if TSE can occur in the soma of larvae by crossing M and *P-1152* females with males which carry an enhancer-trap either in the *scalloped* or *patched* genes and staining imaginal discs in the resulting progeny (*scalloped* and *patched*, but not *P-1152,* are expressed in imaginal discs (data not shown)). Whatever the disc tested (eye, wing, leg, haltere), no repression was detected in the presence of *P-1152* (Figure 2). Thus TSE can occur in third instar larvae and presents the same maternal effect and female germline specificity as in adults.

### TSE silencers can be found in the TAS of various telomeres

Following the discovery of the crucial role of telomeric *P* elements inserted in TAS of the *X* chromosome in P cytotype determination [24], [25], TSE was discovered by using two *P-lacZ* insertions also located at the *X* chromosome telomere and one *P-white-rosy* insertion located at one of the third chromosome telomeres [44]. In each case, the telomeric silencer transgenes were located in TAS. In the same analysis, telomeric transgenes inserted in non-TAS sequences were found to be devoid of repressive capacities. We have extended the search for silencers by testing a number of transgenes located at other telomeres, other centromeres, in euchromatin and on the fourth chromosome. The results are presented in **Table 1**. Transgenes are classified with regard to their genomic location and to their capacity to repress, in *trans*, a homologous euchromatic transgene expressed in the female germline. In addition, data from [51], in which were tested clusters of *P-lacZ-white* transgenes generating variegation in the eye for the *white* marker [54], [55], are also reported. Among 38 insertions located in euchromatin on all chromosomal arms, no transgene was found to be able to induce TSE. In particular, three transgenes located in the “gooseneck”, cytological region 31 located on chromosome *2*, which is bound by HP1 on salivary gland polytene chromosomes, were tested and no silencer was found. Ten transgenes located in pericentromeric heterochromatin from the three main chromosomes were also tested and two transgenes located on the heterochromatic fourth chromosome were tested: no silencer was found.

**Table 1:** Capacity of *P* transgenes to induce *Trans*-Silencing Effect.

	Not Silencer	Silencer
**Telomere**	*SUPor-P690-I* (1A)*, *SUPor-P22-I* (60F)*, *SUPor-P-KG10047* (60F)**, *P-1611* (100F)*, *SUPor-P525-1A* (100F)*, *SUPor-P316-I* (100F)*, *SUPor-P-KG01591* (100F)**	*P-1103* (1A)*^R^, *P-1152* (1A)*^ R^, *SuPor-P863-1* (1A)*, *P-w-y-T2R-PAR* (60F)*, *A4-4* (100F)*^R^, *P-1155* (100F)*
**Centromere**	*P-2004* (20A-B), *SUPor-P-KG01248* (20C*), SUPor-P-KG03740* (20D), *SUPor-P-KG09078* (20D), *CH(2)6* (2R)*, *P-1296* (40A), *P-819* (41A), *P-1784* (80A-F), *P-993* (81F), *P-1695* (81F)	
**Chromosome 4**	*P-6303* (101F), *P-2648* (102F)	
**Euchromatin**	*P-1131* (1C), *P-592* (1E), *P-1164* (2D), *P-1260* (3C), *SUPor-P-KG06450* (7D), *P-1468* (8D), *P-589* (10B), *SUPor-P-KG02704* (11A), *Bl-5536* (12A), *sd-lacZ* (13F), *P-1168* (19A), *P-1085* (19C), *H15-lacZ* (25E), *wg-lacZ* (27F), *SUPor-P-KG08841* (28A), *P-1195* (29C)*, *P-435* (31D)^G^, *P-644* (31D)^G^, *P-476* (31E)^G^, *P-936* (33E), *P-1033* (35D-E), *P-605* (39E)*, *6-2* (50C), *P-1038* (50D), *SUPor-P-KG00786* (52D), *P-2032* (62A-B), *P-1075* (64D), *P-1169* (65C-D), *SUPor-P-KG05833* (68C), *P-1052* (70A), *P-1064* (70F), *P-1173* (84E), *P-0950* (85B), *neur-lacZ* (85C), *P-300* (89B)*, *P-1151* (91B), *SUPor-P-KG10155* (91F), *kay-lacZ* (99B-C)	
**P-lac-w clusters**	*1A-6* (50C, 2 copies), *6-4* (50C, 4 copies), *DX1* (50C, 6 copies), *BX2* (50C, 7 copies), *6-E* (92E, 3 copies)	*T-1* (50C,X-ray, 7 copies)

A large panel of transgenes inserted at various chromosomal locations has been tested for its capacity to induce TSE. Females from lines carrying the tested transgene were crossed with males carrying a *BQ16* or *P-Co1* transgene as target. M females (from the Canton^y^ or *w* ^1118^ strains, devoid of *P* sequences) were crossed with similar males in the same conditions (M control). Overnight *lacZ* staining of ovaries was performed. Tested transgenes were designated as “Silencer” when progeny showed egg chambers with lacZ repression when compared to the M control progeny. Transgenes known to be flanked by TAS are indicated by an asterix; telomeric transgenes inserted in terminal retrotransposons are indicated by two asterixes. Insertions located in the region of the *2L* chromosomal arm which is covered by HP1 on larval polytene chromosomes (called the “gooseneck”) are indicated by ^G^. Among silencers, ^R^ indicates that the effect was described previously in [44]; *P-1155* was not found to be a repressor in the latter study, whereas we identified it as a silencer possibly because we used a more sensitive target transgene. Tested transgenes are *P-lacZ* constructs except for those of the *SUPor-P* series which are *P-white-yellow* and for *P-833* and *P-819* which are *P-white-rosy* constructs. All *SUPor-P* insertions were tested using *P-Co1* as a target so that the two transgenes share long enough sequence homology (*white* marker). The cytological location is given in parenthesis. *P-lac-w* clusters correspond to transgenes in tandem arrays; the number of transgenes is given in parenthesis; X-ray indicates that the line has undergone a chromosomal rearrangement – for details, see [51], 55. Chromosome *1* = **1A**–20F; *2L arm* = **21A**–40F ; *2R arm* = 41A–**60F**; *3L arm* = **61A**–80F; *3R arm* = 81A–**100F**; chromosome *4* = 101A–**102F**; the telomeres are in bold. The properties and references of all transgenes are listed in Table S2. Mapping and orientation of some telomeric insertions inside a TAS repeat are reported in Figure S1.

In contrast, we tested 11 telomeric transgenes located in TAS and found six silencers among these. Three previously undescribed telomeric silencers were found which are located on the first, the second and the third chromosomes. Thus silencers can be located at the telomeres of all main chromosomes. Two transgenes inserted in the LINEs telomeric clusters which are distal to the TAS and which do not present Telomeric Position Effect [41] were tested and did not induce TSE. This confirms thus that the capacity for a transgene to be a TSE silencer requires not only to be at a telomere but also to be inserted specifically in TAS. The only exception to this rule comes from a *P-lacZ-white* transgene cluster called “*T-1”* which is located on the second chromosome. *T-1* results from X-ray treatment of a cluster called *BX2* which contained seven tandem *P-lacZ-white* transgenes located in 50C and which presents weak variegation in the eye. *T-1* presents, on polytene chromosomes, numerous uninterpretable chromosomal rearrangements and presents a very strongly variegating repression of the *white* marker in the eye. This cluster was shown previously to be a strong TSE silencer whereas all the clusters without any rearrangement cannot induce TSE [51]. It is possible that the rearrangements induced by irradiation lead to a sort of “pseudotelomerisation” of this cluster region. It is noteworthy that we did not detect silencers among the 10 centromeric transgenes tested, despite the fact that these transgenes are inserted in heterochromatin.

Finally, an important point is that TSE silencers can have different structures since silencers can be telomeric *P-lacZ*, *P-white-rosy* or *P-white-yellow* transgenes, provided that the target transgene used for the assay exhibits homology with the telomeric insertion. Indeed, telomeric *trans*-repression was shown to be homology-dependent [26]. The present data shows that this homology can result not only from the *lacZ* sequence, but also from the *white* or *rosy* sequences since in some cases, the silencer does not carry *lacZ* and the 0.82kb of *P* element sequence which are common to all these transgenes were previously shown not to be long enough to induce TSE [51]. Thus, silencing of the target likely involves a *cis-*spreading on this target from the sequence homologous to the telomeric transgene to the *lacZ* sequence. In conclusion, the requirements for a transgene to be a TSE silencer are that they be telomeric, flanked by TAS and share homologous sequences with the target. Under these conditions, various telomeres can work and the silencer does not need to carry the repressed gene (*lacZ*). However, not all transgenes inserted in TAS are silencers, a result which can be attributed to a position effect of the transgenes inside the TAS repeat array or to the structure of distal part of the telomere.

### All silencers show the TSE maternal effect

**Figure 3:**
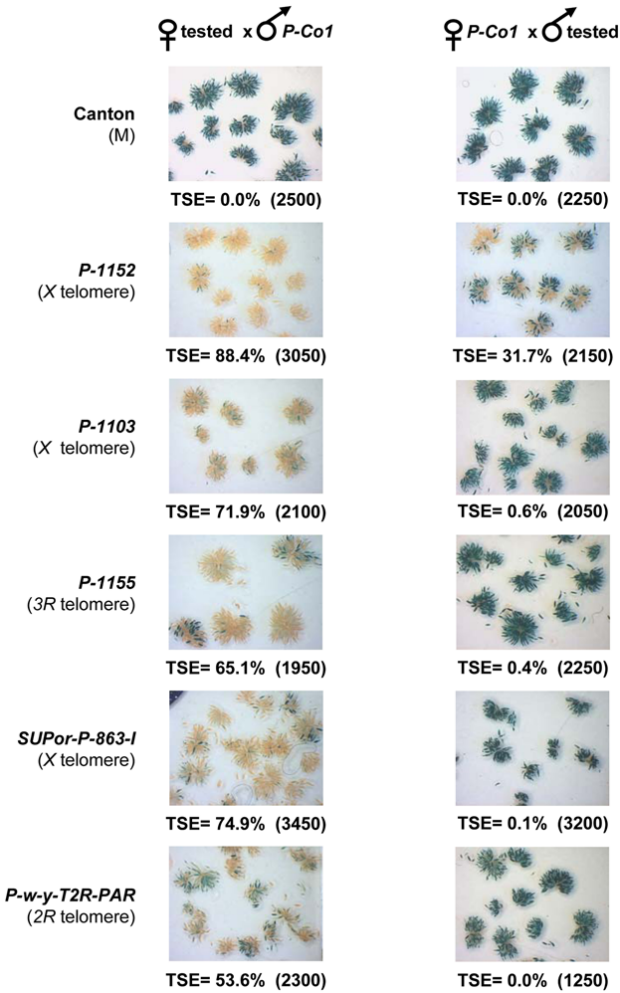
Silencers inserted at different telomeres exhibit a maternal effect. The two reciprocal crosses were performed between individuals carrying the euchromatic *P-otu-lacZ* transgene (*P-Co1*), used as the TSE target, and lines carrying a telomeric silencer transgene inserted in TAS. As an expression control, *P-Co1* individuals were crossed with Canton M individuals (devoid of *P* sequences). In each case, ovaries from G_1_ females were stained overnight for lacZ activity. The percentage of repressed egg chambers (% of TSE) is given with the total number of egg chambers counted in parenthesis. The transgenes tested as silencers are indicated on the figure (on the left) and their structures and locations are described in the text and in Figure S1.

Two main properties are characteristics of TSE. The first one is the maternal effect: strong TSE occurs only when the telomeric silencer is maternally inherited. The second is the variegation: when repression is incomplete a clear cut random “on-off” lacZ staining is observed from one egg chamber to another [46]. This first case of female germline variegation allows an easy quantification of TSE by scoring the repressed egg chambers and determining the percentage of TSE [45]. We tested if the silencers located on different telomeres have the same repression phenotypic properties. Thus, we crossed six telomeric silencers with the *P-Co1* target and measured the TSE in G_1_ females of the two reciprocal crosses **(** **Figure 3** **)**. In each case, a strong maternal effect is observed since a much stronger level of repression is observed when the telomeric transgene is maternally-inherited compared to when it is paternally-inherited. The level of repression appears stronger with silencers which carry two telomeric transgenes (*P-1152*, *SUPor-P-863-I*) than one transgene, a result already described by Roche and Rio using telomeric transgenes located on an *X* minichromosome [44]. In addition, irrespective of the telomeric silencer, variegation among egg chambers is observed when repression is incomplete. Therefore, telomeric transgenes inserted at various telomeres present the same properties with regard to repression in *trans,* strongly suggesting that the same molecular mechanism is involved from one telomere to another.

### TSE functional interaction exists between non homologous telomeres

Next, we tested if silencers located at different telomeres can interact for establishing TSE. TSE establishment was previously shown to require, not only the presence of the chromosomal copy of the telomeric silencer, but also the inheritance of a maternally-transmitted component deposited in the oocyte of females carrying the telomeric silencer [45]. If the female is hemizygous for a silencer, this component can be transmitted independently of the chromosomal copy of the silencer itself. This component has the capacity to stimulate TSE in the progeny provided a copy of this silencer is transmitted by the male. Such a phenomenon makes of TSE a “two component system”, interpreted as resulting from the deposition in the oocyte of small RNAs produced by the telomeric silencer which interact in the zygote at the embryonic state with the chromosomal copy of the silencer in order to render it apt (*via* its heterochromatinization) to establish TSE [45]. We tested if telomeric silencers located at non-homologous telomeres can functionally interact *via* this two component system. In other words, is the maternal component produced by a transgene located on the third chromosome able to stimulate establishment of TSE by telomeric transgenes located on the *X* chromosome and *vice-versa*?

**Figure 4:**
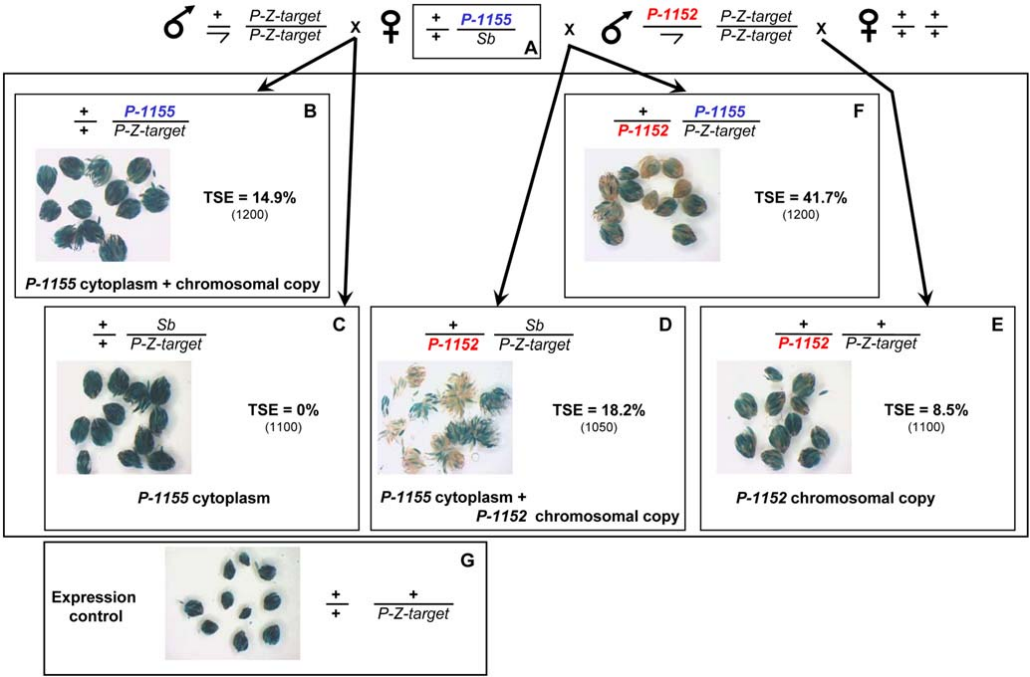
Functional interaction between silencers located in TAS at non-homologous telomeres: a silencer inserted at the *3R* chromosome arm telomere transmits a maternal component which stimulates the repressive properties of a silencer located on the *X*-chromosome telomere. Genotypes are given for chromosomes *1* and *3*. “A” females, hemizygous for the *P-1155* telomeric silencer locus on the third chromosome, were established by crossing homozygous *P-1155* females and males carrying the balancer chromosome *TM3*-*Sb* (marked by the dominant *Stubble* mutation). These “A” females were crossed with males carrying the *P-Co1* euchromatic *P-lacZ* as target in order to recover the “B” and “C” females having inherited, or not, *P-1155*. “A” females were also crossed with *P-1152; P-lacZ-target* males in order to recover the “D” females having inherited *P-1152* from the father and the “F” females having inherited the two telomeric silencers. *P-1152; P-lacZ-target* males were also crossed with females devoid of *P*-transgenes producing “E” females genotypically similar to “D” females, except that they have inherited a naive cytoplasm, whereas, “D” females have inherited a “*P-1155*” cytoplasm. “B–F” females were scored for TSE. “G” females show the expression control for the target. The percentage of repressed egg chambers (% of TSE) is given with the total number of egg chambers counted in parenthesis.

We used two telomeric *P-lacZ* silencers, *P-1152* and *P-1155* which have the same structure but are located on the *X*-chromosome and on the *3R* chromosomal arm, respectively. Females were constructed which had maternally inherited *P-1155* and which had a dominant marker (Sb) on the homologous chromosome *3* (balancer chromosome). **Figure 4** shows that crossing these hemizygous females (“A” females) with males carrying a target transgene produced control “B” females which inherited, from their mother, both the cytoplasm and a chromosomal copy of the telomeric silencer: in these females TSE is about 15%. However, sisters having inherited the *Sb* chromosome do not show any repression (“C” females, TSE = 0%). Thus, the cytoplasm of a *P-1155* female without a chromosomal *P-1155* copy cannot induce TSE. Crossing *P-1152* ; *P-Z-*target males with females devoid of telomeric silencer produces a non-null but weak repression in the progeny (8.5%), as shown by “E” females, a result consistent with the fact that, given the maternal inheritance of TSE, paternal transmission of a telomeric silencer results in weak TSE (see Figure 3 and [51]). Finally, crossing “A” females with males carrying a *P-1152* telomeric silencer and a target transgene allows recovery of females having maternally inherited only a “*P-1155”* cytoplasm and paternally inherited a *P-1152* chromosomal silencer. In that case, significant repression is observed (“D” females, 18% TSE; the difference with the level of E females is highly significant, χ^2^ = 44.4, df = 1, *p*<10^−3^). Thus the *P-1155* cytoplasmic component (incapable by itself of inducing TSE, as shown with “C” females) combined to a paternally inherited *P-1152* telomeric silencer can establish TSE. When the two telomeric silencers were inherited a stronger repression was observed (“F” females, 42% TSE). Thus cytoplasm produced by a female carrying *P-1155* is able to stimulate the repression capacities of a *P-1152* chromosomal copy.

**Figure 5:**
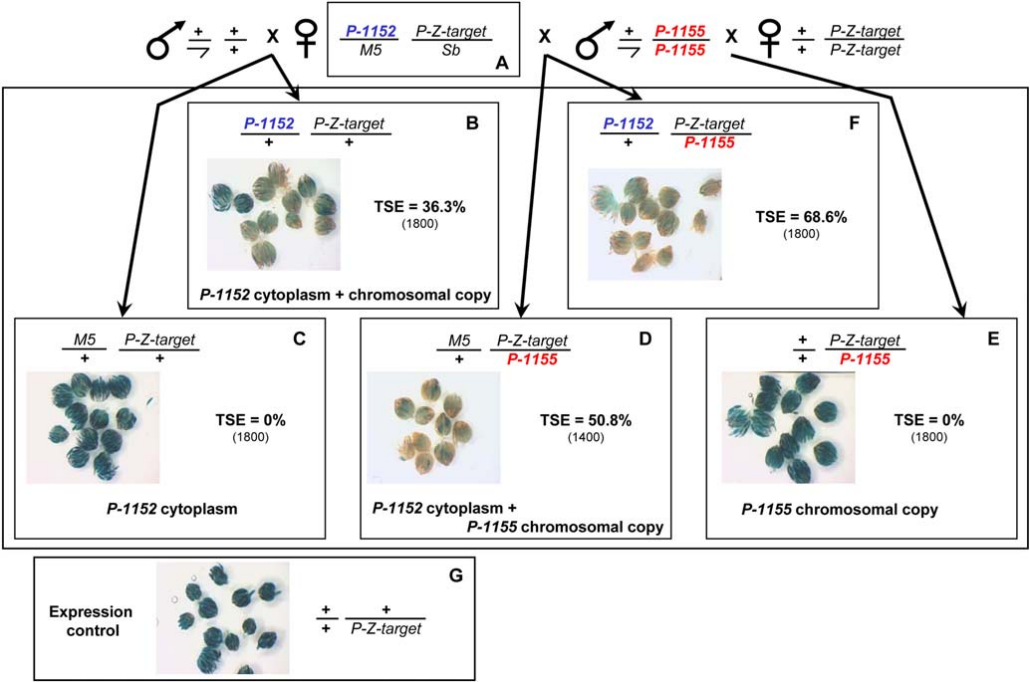
Functional interaction between silencers located in TAS at non-homologous telomeres: reciprocal interaction between the silencers located at the *X* and *3R* arm telomeres. Presentation is similar to that in Figure 4. “A” females, hemizygous for the *P-1152* telomeric silencer locus on the *X* chromosome and carrying the *P-Co1* euchromatic target, were established by crossing homozygous *P-1152; P-Co1* females and males carrying the balancer chromosomes *Muller-5* (*M5*) and *TM3*. These “A” females were crossed with males devoid of *P*-transgenes in order to recover the “B” and “C” females having inherited, or not, *P-1152*. “A” females were also crossed with *P-1155* males in order to recover the “D” females having inherited *P-1155* from the father and “F” females having inherited the two telomeric silencers. *P-1155* males were also crossed with *P-Co1* females producing “E” females genotypically similar to “D” females, except that they have inherited a naive cytoplasm, whereas, “D” females have inherited a “*P-1152*” cytoplasm. “B–F” females were scored for TSE. “G” females show the expression control for the target.

The reciprocal experiment was performed to test the capacity of *P-1152* cytoplasm to stimulate repression by a *P-1155* paternally-inherited transgene. In that case, the semi-dominant marker for the *X* chromosome was *Bar* on the *M5* balancer chromosome. **Figure 5** shows that the cytoplasm of a *P-1152* female without a chromosomal *P-1152* copy cannot induce TSE (“C” females, 0% TSE). The same situation was found for females which inherited *P-1155* transgenes paternally (“E” females, 0% TSE). In contrast, females which maternally inherited only a “*P-1152”* cytoplasm and paternally inherited a *P-1155* chromosomal silencer show strong repression (“D” females, 51% TSE). “B” females which have maternally inherited a *P-1152* chromosomal copy and cytoplasm show unexpectedly moderate TSE (36%). Again, when the two telomeric silencers were inherited a stronger repression was observed (“F” females, 69% TSE). These two experiments show that the cytoplasm associated to a silencer located at a telomere can positively interact with the chromosomal copy of a silencer located at a different telomere to induce TSE suggesting that the same mechanism is involved by transgenes located at various telomeres for establishing this *trans*-silencing.

### Epigenetic transmission of TSE does not require the presence of the target transgene

**Figure 6:**
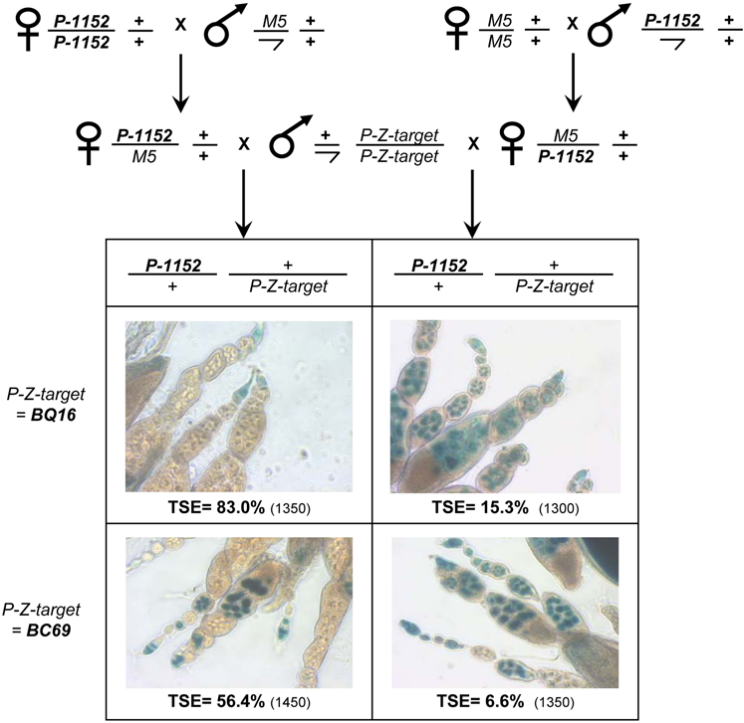
Maternal inheritance of TSE does not require the presence of the target. The two reciprocal crosses were performed between *P-1152* and individuals devoid of *P*-transgenes and carrying the *Muller-5* chromosome marked with *Bar*. G_1_ females have the same genotype, but have inherited different cytoplasms. These G_1_ females were crossed with males carrying an euchromatic *P-lacZ* as target and the capacity of G_2_ females to repress this target was measured after overnight *lacZ* staining. The experiment was performed with two different *P-lacZ* enhancer traps as targets (*BQ16* and *BC69*). The percentage of TSE is given with the total number of egg chambers scored in parenthesis.

TSE exhibits both a maternal effect [51] and a maternal inheritance [45]. The maternal effect is the fact that when performing the reciprocal crosses (female *P-lacZ*-telomeric x male *P-lacZ*-target) and (female *P-lacZ*-target x male *P-lacZ*-telomeric), strong TSE occurs only in the progeny of the first cross. Maternal inheritance is the fact that these two kinds of G_1_ females transmit different repression capacities to their own daughters. So despite the fact that G_2_ females receive a maternally-inherited silencer, they will have different properties because of an epigenetic memory of the properties of their grandmothers. It was shown previously that the effect of the maternal inheritance can be detected for more than five generations conferring to TSE an epigenetic transmission through meiosis [45]. We tested if the maternal inheritance of TSE, previously tested in the presence of the euchromatic target in G_1_ females, can be detected in the absence of the target in G_1_ females, since it could be postulated that the target can itself play a role in the difference between these G_1_ females. For that, we generated the two kinds of G_1_ females which have inherited the *P-1152* silencer, maternally or paternally (**Figure 6**) but which carry no target transgene. We tested further if the maternal inheritance shown previously [45] can be detected. For that, we crossed theses G_1_ females with males carrying a target transgene and measured TSE in G_2_ females. This experiment was performed using two target transgenes (*BQ16* and *BC69*). Figure 6 shows that in both cases a strong difference in TSE level was detected between G_2_ females which have inherited the transgene from their grandmother *vs* their grandfather (83.0% vs 15.3% with *BQ16* and 56.4% *vs* 6.6% with *BC69*). This shows that the two kinds of G_1_ females having the telomeric silencer but not the target transgene can transmit to their progeny different states which play a role in determining the capacity to repress a target. Maternal inheritance of TSE, responsible for its epigenetic behavior, can thus be established in the absence of the target transgene.

### A single telomeric silencer locus can repress two targets located on different chromosomal arms

We finally tested if a single telomeric silencer locus can repress two *P-lacZ* target transgenes inserted at allelic or non-allelic positions. We used the telomeric *P-1152* silencer which carries two copies of *P-lacZ* at the cytological site 1A and the *BQ16* and *P-Co1* target insertions which are located on the *3L* and *3R* chromosomal arms respectively. We measured TSE in females which have inherited a *P-1152* silencer and which are either homozygous for a given target (*BQ16*) or hemizygous for two different targets (*BQ16* and *P-Co1*). **Table 2** shows the crosses performed to generate these genotypes, plus other control genotypes and the levels of TSE measured. The targets expression controls (rows 1–5) show, of course, no repressed egg chambers. The classical TSE positive controls (one silencer locus+one target: rows 6 and 7–92.7% and 89.5%, respectively) show the classical strong levels of TSE found previously for these targets (Figure 3 and [45]). Rows 6 and 9 show that irrespective of the maternal or paternal mode of inheritance of the *BQ16* target, close repression levels are observed (92.7% *vs* 91.5%): thus, in contrast to the mode of inheritance of the telomeric silencer, the mode of inheritance of the target transgene is not crucial for TSE. Rows 10 and 11 show that a maternally-inherited single *P-1152* locus strongly repress two copies of *BQ16* or one copy of *BQ16* plus one copy of *P-Co1*, showing that a single silencer locus can strongly repress two transgenes (89.0% and 78.8% respectively). It is noticeable that the combinations (one silencer+one target) and (one silencer+2 allelic targets) produce similar levels of repression (rows 9 and 10: 91.5% *vs* 89.0%), but this fact is not observed when the two targets are not located on the same chromosomal arm (compare row 11 (78.8%) to rows 9 (91.5%) or 7 (89.5%)). Finally, the level of repression of row 8 (two silencer loci+two allelic targets, 100%) is stronger than the (one silencer loci+two targets) combination (row 9, 91.5%), showing that the number of silencers can influence the level of target repression, a situation already encountered on Figures 4 and 5 using two different silencers (*P-1152* and *P-1155*). In conclusion, a single telomeric silencer can simultaneously induce *trans*-repression at various locations in the genome.

**Table 2:** Capacity of a telomeric locus to repress two target transgenes inserted at allelic or non-allelic positions.

Row	Parental cross	Genotype analysed	% TSE	n
1	♀ *BQ16* **x** ♂ *BQ16*	***+*** **/** ***+*** ** ; ** ***BQ16*** **/** ***BQ16***	**0.0**	1000
2	♀ *P-Co1* **x** ♂ *P-Co1*	***+*** **/** ***+*** ** ; ** ***P-Co1*** **/** ***P-Co1***	**0.0**	1400
3	♀ Canton*^y^* **x** ♂ *BQ16*	***+*** **/** ***+*** ** ; ** ***+*** **/** ***BQ16***	**0.0**	1400
4	♀ Canton*^y^* **x** ♂ *P-Co1*	***+*** **/** ***+*** ** ; ** ***+*** **/** ***P-Co1***	**0.0**	1500
5	♀ *BQ16* **x** ♂ *P-Co1*	***+*** **/** ***+*** ** ; ** ***BQ16*** **/** ***P-Co1***	**0.0**	900
6	♀ *P-1152* **x** ♂ *BQ16*	***P-1152*** **/** ***+*** ** ; ** ***+*** **/** ***BQ16***	**92.7**	1300
7	♀ *P-1152* **x** ♂ *P-Co1*	***P-1152*** **/** ***+*** ** ; ** ***+*** **/** ***P-Co1***	**89.5**	1050
8	♀ *P-1152* ; *BQ16* **x** ♂ *P-1152* ; *BQ16*	***P-1152*** **/** ***P-1152*** ** ; ** ***BQ16*** **/** ***BQ16***	**100**	1085
9	♀ *P-1152* ; *BQ16* **x** ♂ Canton*^y^*	***P-1152*** **/** ***+*** ** ; ** ***BQ16*** **/** ***+***	**91.5**	1450
10	♀ *P-1152* ; *BQ16* **x** ♂ *BQ16*	***P-1152*** **/** ***+*** ** ; ** ***BQ16*** **/** ***BQ16***	**89.0**	1000
11	♀ *P-1152* ; *BQ16* **x** ♂ *P-Co1*	***P-1152*** **/** ***+*** ** ; ** ***BQ16*** **/** ***P-Co1***	**78.8**	1600

The parental cross shown in column 2 was performed at 25°C in order to generate G_1_ females whose genotype is given in column 3 (for chromosomes *1* and *3*). In each case, parental strains carrying transgenes were homozygous for these transgenes. Overnight staining of G_1_ female ovaries was performed and TSE was measured. TSE percentage and the total number of egg chambers counted are given in columns 4 and 5, respectively. *BQ16* and *P-Co1* are both located on chromosome *3,* but are inserted on different chromosomal arms.

## Discussion

### 1 –TSE does not occur in the soma

*Trans*-silencing was tested in various tissues at both the adult and third instar larval stage and no repression was detected in the somatic tissues, nor in the male gonads (Figures 1–2). This result was confirmed with a number of different targets insertions, including some targets which are expressed in both the female germline, the testis and in the soma; in each case the target was sensitive to TSE in the female germline, but not in the soma, nor in the testis (Table S1). This shows that the tissue specificity cannot be attributed to specific properties linked to the genomic context of some of the targets which would render these targets insensitive to TSE. To explain the germline specificity, two main hypotheses can be proposed. The production of the primary small RNAs by the TAS locus would be, for an unknown reason, restricted to the germline. Alternatively, the tissue specificity of TSE could be linked to the amplification step of the piRNA pathway which could be restricted to the germline. Indeed, under the model proposed for this pathway, piRNA biogenesis involves at least three PIWI family proteins: PIWI, AUBERGINE and AGO3 [6], [42]. It has been shown that, whereas PIWI is present in the female germline and in somatic tissues (for example in the follicle cells), AGO3 and AUBERGINE are not detected in the follicle cells, but are present in the germline [6]. Thus, TSE restriction to the germline would result from the fact that the ping-pong positive loop of piRNA biogenesis cannot occur in the soma.

### 2 - Silencers located at different telomeres induce phenotypically similar silencing and interact functionally

TSE induced by silencers located at different telomeres appear to have the same genetic and phenotypic properties and thus likely involve the same mechanism. Indeed, in all cases, an incomplete repression does not lead to an intermediate homogenous pale blue staining of the ovary but to variegation between egg chambers (Figure 3). In addition, the various telomeres can interact. Indeed, TSE establishment was previously shown to require both a chromosomal copy of the telomeric silencer and a maternally transmitted component which can be transmitted independently of the chromosomal telomeric copy itself [45]. This maternal component was interpreted to be small RNAs produced by the telomeric silencer in the female and deposited in the cytoplasm of the oocyte. Maternal deposition of small RNAs was described in *Drosophila virilis* where repression of hybrid dysgenesis, linked to the *Penelope* retroelement, has been correlated to *Penelope* small RNAs deposition in the embryo [56]. In *D. melanogaster*, piRNAs of TAS have been detected for both the TAS of the *X* chromosome [6] and the TAS of the *3R* chromosomal arm telomere [43]. In the case of TSE, maternally-transmitted small RNAs would modify the chromatin structure of the paternally or maternally-inherited chromosomal telomeric copy, rendering it apt to produce small RNAs and to maintain their concentration. Such a positive loop between small RNA production and chromatin structure modifications could explain the epigenetic transmission of TSE over several generations. We show here that this functional interaction can exist between silencers located at non-homologous telomeres (*X* and third chromosome telomeres, Figures 4 and 5). This suggests that the various telomeric TAS platforms would use a similar positive loop pathway. TSE being homology-dependent, a functional interaction between telomeres is possible because of the full sequence homology between the telomeric insertions used (*P-1152* and *P-1155* are the same construct) and, to a lesser extent, the partial homology between various TAS, (especially *X* and *3R* linked TAS). In conclusion, the TAS piRNA-producing platform located at different telomeres can cooperate for establishing repression. Interaction between non-homologous telomeres in *Drosophila* was also shown to exist for Telomeric Position Effect (TPE), although in this case it has been proposed to involve pairing between different telomeres [57]. At the level of *P* element repression, interactions between non-homologous telomeres for TSE shows that in natural populations, the *P* elements which are frequently present not only on the *X* chromosome telomere [58], but also on autosomal telomeres (data not shown) can cooperate to establish the P cytotype. This hypothesis is also illustrated by the fact that a transgene located at the third chromosome telomere can stimulate *P* repression establishment by regulatory *P* elements located on the *X* chromosome [59]. Natural telomeric *P* elements located at different telomeres in natural populations can thus cooperate to establish the P cytotype. However, not all telomeric transgenes inserted in TAS are TSE silencers (Table 1), a result which can be attributed to variations in the position of these transgenes inside the TAS tandem array, to the length of this array or to the structure of the retrotransposon array distal to the TAS [60]. Consistent with this hypothesis, telomere structure was shown to affect TPE significantly [36], [38], [57]. In addition, fragments of transgenes generated by TAS region rearrangements which escape detection by PCR analysis may also play a role in the repressive capacities of the telomeric transgenes.

### 3 – TSE silencers appears restricted to telomeric sites but can repress targets located anywhere in the genome

In TSE studies reported so far, telomeric transgenes, but not centromeric transgenes, although also being heterochromatic, were found to be silencers and all euchromatic *P-lacZ* insertions expressed in the female germline were found to be targets (Tables 1 and S1) [26], [44]. Indeed, nineteen targets located on chromosomes *1*, *2* and *3* were tested and all were sensitive to *trans*-repression. We found thus no TSE escaper, even though the sensitivity of the different targets to repression may vary. For example *BC69*, located on the second chromosome, almost never undergoes complete repression, whereas *BQ16* or *P-Co1* can show nearly complete repression covering all stages of oogenesis. This sensitivity does not appear to be correlated to the level of expression of the target (data not shown).

How can the fact that we did not find TSE silencers located in centromeric heterochromatin be explained? Three main models have been proposed to explain the molecular mechanism of target repression by the silencer [46]. Two models involve recognition of the target(s) by the silencer sequence at the DNA level upon scanning of the genome by telomeric sequences. This scanning would lead to pairing of the two homologous sequences. In a first model, this pairing would result in *trans*-heterochromatinization of the target induced by the telomeric sequence which is itself heterochromatinized. A second model would involve dragging of the target to a compartment of the nucleus where subtelomeric heterochromatin would be localized. In a third model, telomeric sequences would produce small non coding RNAs which would result in silencing of the target: this last phenomenon could occur either *via* degradation of the RNA produced by the target, or *via* induction of target heterochromatinization due to interaction of the smalls RNAs produced by the telomeric silencer with the nascent transcripts of the target [61], [62]. To explain that telomeric, but not centromeric insertions, can be silencers, it is possible to propose, taking into consideration the first two models, that scanning of the genome is a property of the telomeres and not of the centromeres. Under the third model, it is possible that, in the germline, the telomeric TAS locus produces small RNAs but not the centromeric loci, at least those which are close to the centromeric *P-lacZ* insertions we tested. This perhaps seems contradictory to the fact that the other known master site of TEs control in *Drosophila* is centromeric. Indeed, the *flamenco* locus regulating *gypsy* and the *COM* locus regulating *ZAM* and *Idefix* are located close to the centromere of the *X* chromosome. However, *flamenco* and *COM* exert their repression in somatic follicle cells, the starting points of the transposition events of these retrotransposons [63]–[68]. It remains thus to be demonstrated that *flamenco* and *COM* have a direct repressional capacity in the germline. However, it also remains possible that centromeric TSE silencers exist but that being rare we did not detect them in our screen. A greater number of centromeric insertions therefore needs to be tested before we can conclude this point more categorically. Finally, the fact that a single telomeric silencer locus can repress two targets on different chromosomal arms is more consistent with the third model in which the crucial point is the concentration of piRNAs present in the nucleus. According to this model, if this concentration is above a certain threshold, all targets located at any genomic site could be simultaneously repressed. Regarding *P* element repression, it is possible to propose that numerous euchromatic copies of natural *P* elements are repressed by a single telomeric *P* element.

### 4 – A telomeric silencer alone can establish epigenetic maternal inheritance

TSE transmission over generations shows a maternal inheritance whose effect can be detected for more than five generations [45]. Indeed, the female progeny of the reciprocal crosses (female *P-lacZ*-telomeric; *P-lacZ*-target x male M) and (female M x male *P-lacZ*-telomeric; *P-lacZ*-target), not only has different silencing properties with regard to their own ovaries, but will transmit different silencing properties to G_2_ females, despite that these two types of G_2_ females have inherited the telomeric silencer from a female. The G_1_ dissymmetry is thus the starting point of epigenetic transmission of TSE over generations. The question now is whether the presence of the target transgene is necessary for the dissymmetry in transmission from G_1_ to G_2_ or, alternatively, if the telomeric silencer alone is able to establish it. In TSE, the influence of the maternal inheritance is interpreted to be linked to the amount of piRNAs maternally-transmitted from the female to the progeny. According to the ping-pong model, two partners are involved in the biogenesis of piRNAs: the master regulatory locus which carries sequences of the repressed transposable element and the target element locus (the euchromatic TEs copies) [6]. The master regulatory locus produces mainly antisense TEs RNAs and the euchromatic copies mainly sense RNAs. The ping-pong model proposes that small antisense piRNAs associated with AUBERGINE or PIWI interact with sense RNAs produced by target TEs copies and cleave it, in order to induce the production of small sense piRNAs associated with AGO3, which in turn will interact with antisense transcripts produced by the master locus of repression. In that system, the euchromatic copy appears necessary for the ping-pong interaction to take place and to increase the concentration of piRNAs. We show here that in TSE, females issued from the two reciprocal crosses (female *P-lacZ*-telomeric x male M) and (female M x male *P-lacZ*-telomeric) have different TSE capacities indicating that maintenance of the dissymmetry in repression capacity in G_1_ females does not require the presence of target elements (Figure 6). This suggests that the telomeric silencers can produce all the components required, not only to establish the repression equilibrium state (inside the *P-1152* line), but also to induce its epigenetic maternal inheritance when out-crossing. Consequently, if TSE involves the ping-pong model [6], this suggests that the telomeric silencer locus can produce both the sense and anti-sense transcripts involved in piRNA biogenesis.

### 5- TSE: a major component of the P cytotype elicited by telomeric *P* elements

TSE, a repression mechanism shown to exist by the use of *P*-transgenes, is likely a major component of *P* element repression elicited by natural telomeric *P* elements. Indeed, inheritance of the TSE repressive capacities over generations has the same epigenetic behavior as the P cytotype [22], [45], [47], [69], [70]. Further, both TSE and the repression established by telomeric *P* elements are sensitive to mutations affecting HP1 and the *piwi*-family protein AUBERGINE [25], [30], [31]. At *Drosophila* telomeres, both defective and complete *P* elements can be found in TAS [25], [26], [32]. In the case of defective telomeric *P* elements, repression should occur *via* only TSE between these telomeric *P* copies, unable to encode a repressor, and the targets which are euchromatic autonomous copies of *P* elements. In contrast, in the case of autonomous telomeric *P* elements, both TSE and production of *P*-encoded repressor can be supposed to occur.

But is TSE the only component of the P cytotype established by telomeric *P* elements? Under such a hypothesis, the tissue specificity associated with TSE should show the same characteristics as that of *P* repression by telomeric *P* elements in TAS. In fact, the tissue specificities of the two phenomena are partially overlapping. TSE and P cytotype by telomeric *P* elements are both restricted to the germline. Indeed, the three *X*-chromosome telomeres containing regulatory *P* elements we isolated (*Lk-P*(1A) from Russia, *Ch-P*(1A) from France and *NA-P*(1A) from Tunisia) were shown to have weak or null repression capacities in the somatic tissues [24]–[26]. Lack of somatic repression capacities was found for the two other natural telomeric regulatory *P* elements (called *TP5* and *TP6*) deriving from American populations [23], [32]. In contrast, TSE is detected only in the female germline, as tested with several different *P-lacZ* targets (Table S1), whereas repression by telomeric natural *P* elements can be detected in both sexes. Indeed, repression was detected in the female germline with all the telomeric natural *P* elements described above, but repression was also found in males when tested. *Lk-P*(1A) was shown to repress *P*-element excision in the male germline (using a *P-white* transgene excision assay) [24], but this line carries two telomeric autonomous *P* elements which can encode a repressor. In contrast, the *TP5* and *TP6* elements correspond to defective telomeric *P* elements inserted in TAS at the *X* chromosome [32] and induce *P*-repression in males, as tested with dysgenic sterility (atrophy of the testis) assay and *P* element excision assay (using an hypermutable *P*-induced allele called *sn* ^w^) [23], [32]. GD repression in males can be explained by the developmental stage at which dysgenic sterility is determined. Indeed, gonadal dysgenesis is determined during early development at late embryonic stages and reflects simply a maternally-transmitted property (deposition of maternal *P* repressive factors in the cytoplasm of the oocyte) [30], [47]. In contrast, the occurrence of repression of *P*-element excision in the male germline by the *TP5* and *TP6* elements, as tested with the *sn* ^w^ assay, is more striking, since this assay takes place in the adult testis and transmission of the maternal component alone is not sufficient to repress *P* excision at the adult stage [24], [32]. The defective *TP5* and *TP6* elements have been shown to encode a polypeptide devoid of repressive capacities [71]. They are thus thought to repress *P* element activity *via* TSE, suggesting that TSE can work in the male adult germline. Two hypotheses can be proposed to explain this discrepancy: 1- TSE may occur only in a limited subset of germline cells of the testis rendering detection of *lacZ* staining difficult; 2- TSE does not occur in the testis and the *TP5* and *TP6* telomeric elements repress *P* element excision in the adult male germline by another mechanism than TSE. However, the lack of effect of TSE in the male gonads at the third instar larvae (Figure 2) is more consistent with the second hypothesis. In any case, it is clear that TSE can play a role in *P* element repression in females at all stages and in males at least in embryos to establish protection against GD sterility.

In conclusion, following its arrival in the *D. melanogaster* genome, the *P* elements inserted both at telomeres into TAS and at various sites in euchromatin. Euchromatic *P* elements increased copy number (30–40) over several generations (25–40) before repression occurred as shown by transformation experiments of M lines with the complete *P* element [27]–[29]. In contrast, *P* elements inserted in TAS, provided a strong repression mechanism which is elicited by a small number of copies (1–2). Telomeric *P* elements thus likely had a pivotal role in P cytotype establishment in *Drosophila* natural populations, a result consistent with the fact that telomeric *P* elements can be found in natural populations geographically widespread [25], [32], [58].

## Supporting Information

Table S1 Trans-Silencing Effect targets: P-lacZ transgenes tested for their capacity to be repressed by a telomeric silencer. Males from lines carrying the P-lacZ transgene, tested as target, were crossed with females carrying the P-1152 telomeric silencer and with females devoid of P-transgenes (Cantony or w1118 M lines) as a control for target transgene expression. Overnight lacZ staining of ovaries and testis were performed. Tested transgenes were designated as “target” when lacZ expression was repressed in the presence of P-1152 when compared to the M control. All transgenes tested showing expression in the female germline (nurse cells and/or mature oocyte) were repressed in this tissue by P-1152, whereas P-1152 never showed any repression capacity in the somatic follicle cells with any target transgene. For example, in the case of transgenes expressed in both the female germline and the soma (P-1039, ABOO, P-1061), repression was observed in the female germline, but not in the soma. In the case of P-0321, the transgene corresponds to an hedgehog enhancer trap expressed in the somatic terminal filament. It is not sensitive to TSE. In the female germline, TSE can occur at all stages of oogenesis, as shown for example with BQ16 of BC69 which are expressed at all stages (from germarium to mature oocytes). No repression by P-1152 was detected with target transgenes expressed in the testis. The name of the transgene is given with the cytological location, when known, between parenthesis. (1A–20F, chromosome 1; 21A–60F, chromosome 2; 61A–100F, chromosome 3). The strains referred to as P-nnnn were obtained from the Bloomington Stock Center and have been renamed later to as #1nnnn by the stock center (for example, P-1039 was renamed 11039). Some of the strains have been discarded from the stock center. The properties and references of all transgenes are listed in Table S2.
(0.02 MB RTF)

Table S2 Genotype and references of transgene(s) insertions tested as TSE silencers or targets. (A) Name of the insertion used in the present study; (B) Transgene(s) location on salivary glands polytene chromosomes; (C) Insertion genotype; (D) Transgene(s) name as referenced in flybase; (E) Flybase ID of the insertion; (F) Reference describing the insertion; (G) Bloomington stock number presently used (if any). Further informations concerning the transgene structure are as follows: (Transgene construct name/Transgene Flybase ID/Transgene reference): (P{lacW}/FBtp0000204/[33]); (P{PZ}/FBtp0000210/[22]); (P{wAR}/FBtp0000064/[2]); (P{wA}/FBtp0000063/[2]); (P{PLH}/FBtp0003686/[34]); (P{GT1}/FBtp0002720/[35]); (P{A92}/FBtp0000154/[36]); (P{otu-lacZ.Co}/FBtp0015417/[8]); (P{SUPor-P}/FBtp0001587/[37]); (P{HZ}/FBtp0000211/[38]); (P{lArB}/FBtp0000160/[39]). Flybase: http://flybase.bio.indiana.edu/. (See the additional file called “Table References S2”)
(0.31 MB RTF)

Figure S1 Position inside the TAS sequence of telomeric transgenes in relation to their repression capacities. All the transgenes analyzed here have been found to be localized inside a 173bp TAS subrepeat, a motif tandemly repeated (3–4 times) inside each TAS unit [1]. This 173bp motif was also found to be tandemly repeated in the TAS described for other telomeres (2R and 3R chromosomal arms) [2]. Transgenes therefore are drawn along a single 173bp subrepeat. For each transgene, the name, the orientation (arrowhead indicates the 3′ P element end) and the chromosomal arm (indicated by the color) is given. TSE silencer transgenes are positioned above the line whereas transgenes devoid of repression capacities are positioned below it. Both P-1152 and SUPor-P-863-I were found to carry two transgene copies at the same positions. The P-1152 copies are located at the same position in two adjacent TAS 173bp subrepeats and result likely from a 173bp subunit duplication. SUPor-P-863-I has a tandem repeat of two SUPor-P copies in direct orientation, this tandem being flanked by a target site duplication: this results likely from a transgene rearrangement which derived from a single initial transgene insertion. These copies are numbered 1 and 2 and are shown at the same position. A4-4 was described and mapped previously [2]–[6]. (See the additional file called “Figure Notes and References S1”).
(0.38 MB TIF)

Figure Notes and References S1 Notes and references for Figure S1
(0.06 MB RTF)

Table References S2 References for Table S2
(0.05 MB RTF)
