# Supplementary material for: Telomeric Trans-Silencing in Drosophila melanogaster: Tissue Specificity, Development and Functional Interactions between Non-Homologous Telomeres
Source: PLoS One. 2008 Sep 22;3(9):e3249. doi: 10.1371/journal.pone.0003249 (PMC2547894; doi:10.1371/journal.pone.0003249)
Supplement: Table S1 — Trans-Silencing Effect targets: P-lacZ transgenes tested for their capacity to be repressed by a telomeric silencer. Males from lines carrying the P-lacZ transgene, tested as target, were crossed with females carrying the P-1152 telomeric silencer and with females devoid of P-transgenes (Cantony or w1118 M lines) as a control for target transgene expression. Overnight lacZ staining of ovaries and testis were performed. Tested transgenes were designated as “target” when lacZ expression was repressed in the presence of P-1152 when compared to the M control. All transgenes tested showing expression in the female germline (nurse cells and/or mature oocyte) were repressed in this tissue by P-1152, whereas P-1152 never showed any repression capacity in the somatic follicle cells with any target transgene. For example, in the case of transgenes expressed in both the female germline and the soma (P-1039, ABOO, P-1061), repression was observed in the female germline, but not in the soma. In the case of P-0321, the transgene corresponds to an hedgehog enhancer trap expressed in the somatic terminal filament. It is not sensitive to TSE. In the female germline, TSE can occur at all stages of oogenesis, as shown for example with BQ16 of BC69 which are expressed at all stages (from germarium to mature oocytes). No repression by P-1152 was detected with target transgenes expressed in the testis. The name of the transgene is given with the cytological location, when known, between parenthesis. (1A–20F, chromosome 1; 21A–60F, chromosome 2; 61A–100F, chromosome 3). The strains referred to as P-nnnn were obtained from the Bloomington Stock Center and have been renamed later to as #1nnnn by the stock center (for example, P-1039 was renamed 11039). Some of the strains have been discarded from the stock center. The properties and references of all transgenes are listed in Table S2. (0.02 MB RTF) [file pone.0003249.s001.rtf]

Ovary germline expression: repressed	Ovary somatic expression: not repressed	Testis expression: not repressed	
R3-29 (1A-B), P-0554 (3F), P-1167 (6D-E),        ABOO (23B-C), P-2225 (32C), BC69 (35B-C), BL9402 (46E), P-1039 (60B), P-1206 (61A),             P-1124 (62A), BQ16 (64C), P-0587 (66B),               P-1153 (71A-B), P-Co1 (87A-B), H162 (87B),         DY112 (83F), P-1002 (96F), P-1061 (100D),          PLH3 (autosome).
	ABOO (23B-C), P-1038 (50D), P-1039 (60B),              P-2032 (62AB), BA37 (87F), P-0321 (94E),                P-1061 (100D) 
	ABOO (23B-C), BC69 (35B-C),                    P-1039 (60B), BQ16 (64C), P-Co1 (87A-B), PLH3 (autosome)
	

Table S1. Trans-Silencing Effect targets: P-lacZ transgenes tested for their capacity to be repressed by a telomeric silencer.
