# Supplementary material for: Telomeric Trans-Silencing in Drosophila melanogaster: Tissue Specificity, Development and Functional Interactions between Non-Homologous Telomeres
Source: PLoS One. 2008 Sep 22;3(9):e3249. doi: 10.1371/journal.pone.0003249 (PMC2547894; doi:10.1371/journal.pone.0003249)
Supplement: Table S2 — Genotype and references of transgene(s) insertions tested as TSE silencers or targets. (A) Name of the insertion used in the present study; (B) Transgene(s) location on salivary glands polytene chromosomes; (C) Insertion genotype; (D) Transgene(s) name as referenced in flybase; (E) Flybase ID of the insertion; (F) Reference describing the insertion; (G) Bloomington stock number presently used (if any). Further informations concerning the transgene structure are as follows: (Transgene construct name/Transgene Flybase ID/Transgene reference): (P{lacW}/FBtp0000204/[33]); (P{PZ}/FBtp0000210/[22]); (P{wAR}/FBtp0000064/[2]); (P{wA}/FBtp0000063/[2]); (P{PLH}/FBtp0003686/[34]); (P{GT1}/FBtp0002720/[35]); (P{A92}/FBtp0000154/[36]); (P{otu-lacZ.Co}/FBtp0015417/[8]); (P{SUPor-P}/FBtp0001587/[37]); (P{HZ}/FBtp0000211/[38]); (P{lArB}/FBtp0000160/[39]). Flybase: http://flybase.bio.indiana.edu/. (See the additional file called “Table References S2”) (0.31 MB RTF) [file pone.0003249.s002.rtf]

(A)                             Line name

	(B)                          Cytological location
	(C)                                                                                         Insertion genotype

	(D)         Transgene structure
	(E)              Insertion flybase ID
	(F) Insertion reference
	(G)              Bloomington stock         number	
6-2	50C 	P{w[+mC]=lacW}50C	P{lacW}	FBti0016763	[1]	-	
6-4	50C 	P{w[+mC]=lacW}50C.x4	P{lacW}	FBti0016765	[1]	-	
1A-6	50C 	P{w[+mC]=lacW}50C.x2	P{lacW}	FBti0016762	[1]	-	
6-E	92E	P{w[+mC]=lacW}92E.x3	P{lacW}	FBti0012449	[1]	-	
A4-4	100F 	P{wA}4-4	P{wA}	FBti0001478	[2]	-	
ABOO	23B-C	P{A92}fs(2)AB00[AB00]	P{A92}	FBti0003393	[3]	-	
BA37	87F 	P{A92}fs(2)BA37[BA37]   	P{A92}	FBti0003418	[4]	-	
BC69	35B-C	P{A92}vas[BC69]	P{A92}	FBti0003420	[4]	-	
Bl-5536	12A	P{ry[+t7.2]=lArB}12A8	P{lArB}	FBti0009136	[5]	5536	
BL9402	46E	P{ry[+t7.2]=lArB}14-3-3zeta[P1188]/CyO	P{lArB}	FBti0009360	[6]	9402	
BQ16	64C 	P{A92}fs(3)BQ16[BQ16]	P{A92}	FBti0003435	[4]	-	
BX2	50C 	P{w[+mC]=lacW}50C.x7	P{lacW}	FBti0016766	[1]	-	
CH(2)6	2R h42-h44	P{ry[+t7.2]=PZ}CH(2)6    	P{PZ}	FBti0003833	[7]	-	
DX1	50C 	P{w[+mC]=lacW}50C.x6	P{lacW}	FBti0022549	[8]	-	
DY112	83F 	P{lacW}S073214a, l(3)S073214b[S073214b]/TM3	P{lacW}	FBti0022877	[9]	-	
H15-LacZ	25E	P{ry[+t7.2]=PZ}H-15 	P{PZ}	FBti0002922	[10]	-	
H162	87B	P{ry[+t7.2]=HZ}svp[3]/TM3	P{HZ}	FBti0002862	[11]	7314	
kay-lacZ	99B-C	P{w[+mC]=lacW}kay[S135103]    	P{lacW}	FBti0018172	[12]	-	
neur-lacZ	85C 	P{ry[+t7.2]=lArB}neur[A101]/TM3	P{lArB}	FBti0002306	[13]	4369	
ptc-lacZ	44D-E	P{A92}ptcH84	P{A92}	FBti0002696	[14]	-	
P-0321	94E	P{ry[+t7.2]=PZ}hh[rJ413] CG31457[rJ413]/TM3	P{PZ}	FBti0002944	[15]	10321	
P-0554	3F 	P{ry[+t7.2]=lArB}Vap-33-1[47]	P{lArB}	FBti0005686	[16]	10554	
P-0587	66B	P{ry[+t7.2]=lArB}99	P{lArB}	FBti0005687	[16]	-	
P-0950	85B	P{ry[+t7.2]=lArB}A119.1M3/TM3	P{lArB}	FBti0005694	[16]	10950	
P-1002	96F 	P{ry[+t7.2]=lArB}A353.2M3/TM3	P{lArB}	FBti0005718	[16]	11002	
P-1033	35D-E	CyO, P{ry[+t7.2]=lArB}A507.2M2, chif[A507]/l(2)*[*]	P{lArB}	FBti0003389	[16]	11033	
P-1038	50D	CyO, P{ry[+t7.2]=lArB}A4.1M2/ Adh[*] cn[*] l(2)*[*]	P{lArB}	FBti0004638	[16]	-	
P-1039	60B	P{ry[+t7.2]=PZ}eIF-5A[01296]/CyO	P{PZ}	FBti0005186	[17]	11039	
P-1052	70A	P{ry[+t7.2]=lArB}A77.1M3	P{lArB}	FBti0005736	[16]	-	
P-1061	100D	P{ry[+t7.2]=lArB}l(3)B14.1M3[1]/TM3	P{lArB}	FBti0004874	[16]	11061	
P-1064	70F 	P{ry[+t7.2]=lArB}B21.1M3 	P{lArB}	FBti0004639	[16]	-	
P-1075	64D	P{ry[+t7.2]=lArB}B72.1M3	P{lArB}	FBti0004634	[16]	-	
P-1085	19C 	P{ry[+t7.2]=lArB}A317.1F1	P{lArB}	FBti0005714	[16]	-	
P-1103	1A	P{ry[+t7.2]=lArB}A461.1F1	P{lArB}	FBti0005726	[16]	-	
P-1124	62A	P{ry[+t7.2]=lArB}l(3)A519.1F3[1]	P{lArB}	FBti0004873	[16]	11124	
P-1131	1C 	P{ry[+t7.2]=lArB}A168.1F1	P{lArB}	FBti0004635	[16]	-	
P-1151	91B	P{ry[+t7.2]=lArB}A256.2F3/TM3	P{lArB}	FBti0005705	[16]	11151	
P-1152	1A	P{ry[+t7.2]=lArB}A171.1F1	P{lArB}	FBti0005700	[16]	-	
P-1153	71A-B	P{ry[+t7.2]=lArB}A179.4F3/TM3	P{lArB}	FBti0005701	[16]	11153	
P-1155	100F 	P{ry[+t7.2]=lArB}A102.2F3	P{lArB}	FBti0005691	[16]	-	
P-1164	2D	P{ry[+t7.2]=lArB}A297.1F1	P{lArB}	FBti0005709	[16]	-	
P-1167	6D-E	P{ry[+t7.2]=lArB}A522.2F1	P{lArB}	FBti0005732	[18]	-	
P-1168	19A	P{ry[+t7.2]=lArB}A135.2F1	P{lArB}	FBti0005697	[16]	-	
P-1169	65C-D	P{ry[+t7.2]=lArB}A131.1F3/TM3	P{lArB}	FBti0005696	[16]	11169	
P-1173	84E	P{ry[+t7.2]=lArB}puc[A251.1F3]/TM3	P{lArB}	FBti0005134	[19]	11173	
P-1195	29C 	P{w[+mC]=lacW}emb[k16715]/CyO    	P{lacW}	FBti0006347	[20]	11195	
P-1206	61A	P{ry[+t7.2]=lArB}A55.1M3	P{lArB}	FBti0005733	[16]	-	
P-1260	3C 	P{ry[+t7.2]=lArB}A418.1F1	P{lArB}	FBti0005724	[16]	-	
P-1296	40A	P{ry[+t7.2]=lArB}A229.1F1	P{lArB}	FBti0005704	[21]	11296	
P-1468	8D	P{ry[+t7.2]=lArB}lz[A27.1F1]	P{lArB}	FBti0003703	[16]	11468	
P-1611	100F 	P{ry[+t7.2]=PZ}MI02[03847]	P{PZ}	FBti0005492	[20]	-	
P-1695	81F 	P{ry[+t7.2]=PZ}06713	P{PZ}	FBti0005565	[20]	-	
P-1784	80A-F	P{ry[+t7.2]=PZ}ms(3)80[03817]/TM3   	P{PZ}	FBti0003533	[22]	11784	
P-2004	20A-B	P{w[+mC]=lacW}su(f)[G0393]/FM7c    	P{lacW}	FBti0013902	[23]	12004	
P-2032	62A-B	P{ry[+t7.2]=lArB}A100.1M3	P{lArB}	FBti0005690	[16]	-	
P-2225	32C 	P{ry[+t7.2]=PZ}piwi[06843]/CyO	P{PZ}	FBti0004305	[5]	12225	
P-2648	102F 	P{w[+mGT]=GT1}activin-beta[BG01941]	P{GT1}	FBti0017661	[24]	-	
P-300	89B	P{w[+mC]=lacW}l(3)L1820[L1820]/TM3	P{lacW}	FBti0009921	[20]	10300	
P-435	31D	P{w[+mC]=lacW}nmd[k10909]/CyO    	P{lacW}	FBti0006021	[20]	10435	
P-476	31E	P{w[+mC]=lacW}KdelR[k00311]/CyO   	P{lacW}	FBti0006932	[20]	10476	
P-589	10B	P{ry[+t7.2]=lArB}112	P{lArB}	FBti0005679	[16]	-	
P-592	1E	P{ry[+t7.2]=lArB}175	P{lArB}	FBti0005682	[16]	-	
P-605	39E	P{w[+mC]=lacW}l(2)k06113[k06113]/CyO    	P{lacW}	FBti0006756	[20]	10605	
P-6303	101F 	P{w[+mC]=lacW}ci[Dplac]   	P{lacW}	FBti0002268	[25]	6303	
P-644	31D	P{w[+mC]=lacW}RnrL[k06709]/CyO   	P{lacW}	FBti0006728	[20]	10644	
P-819	41A	P{w[+tAR] ry[+t7.2AR]=wA[R]}41A	P{wAR}	FBti0001324	[26]	10819	
P-936	33E	P{ry[+t7.2]=PZ}bun[00255]/CyO	P{PZ}	FBti0005165	[27]	10936	
P-993	81F 	P{ry[+t7.2]=lArB}A321.3M3/TM3	P{lArB}	FBti0005715	[16]	10993	
P-Co1	87A-B	P{otu-lacZ.Co}1	P{otu-lacZ.Co}	FBti0022550	[8]	-	
PLH3	Autosomal	P{PLH}3	P{PLH}	FBti0016655	[28]	-	
R3-29	1A-B	P{w[+mC]=lacW}R3-29-2	P{lacW}	FBti0022444	[8]	-	
sd-lacZ	13F 	P{ry[+t7.2]=lArB}sd[ETX4]	P{lArB}	FBti0005141	[29]	5150	
SUPor-P-22-1	60F 	P{y[+mDint2] w[BR.E.BR]=SUPor-P}22-1	P{SUPor-P}	-	[30]	-	
SUPor-P-316-I	100F 	P{y[+mDint2] w[BR.E.BR]=SUPor-P}316-1	P{SUPor-P}	FBti0074111	[31]	-	
SUPor-P-525-1A	100F 	P{y[+mDint2] w[BR.E.BR]=SUPor-P}525-1A	P{SUPor-P}	FBti0074084	[32]	 	
SUPor-P-690-1	1A	P{y[+mDint2] w[BR.E.BR]=SUPor-P}690-1	P{SUPor-P}	-	[32]	-	
SUPor-P-863-1	1A	P{y[+mDint2] w[BR.E.BR]=SUPor-P}863-1	P{SUPor-P}	-	[32]	-	
SUPor-P-KG00786	52D	P{y[+mDint2] w[BR.E.BR]=SUPor-P}KG00786	P{SUPor-P}	FBti0018620	[24]	12918	
SUPor-P-KG01248	20C 	P{y[+mDint2] w[BR.E.BR]=SUPor-P}CG17601[KG01248]	P{SUPor-P}	FBti0021679	[24]	13713	
SUPor-P-KG01591	100F 	P{y[+mDint2] w[BR.E.BR]=SUPor-P}KG01591   	P{SUPor-P}	FBti0074101	[31]	-	
SUPor-P-KG02704	11A	P{y[+mDint2] w[BR.E.BR]=SUPor-P}KG02704	P{SUPor-P}	FBti0018631	[24]	12885	
SUPor-P-KG03740	20D	P{y[+mDint2] w[BR.E.BR]=SUPor-P}CG9561[KG03740]   	P{SUPor-P}	FBti0021784	[24]	13798	
SUPor-P-KG05833	68C 	P{y[+mDint2] w[BR.E.BR]=SUPor-P}CG6175[KG05833]/TM3	P{SUPor-P}	FBti0024937	[24]	15102	
SUPor-P-KG06450	7D	P{y[+mDint2] w[BR.E.BR]=SUPor-P}KG06450	P{SUPor-P}	FBti0024566	[24]	14489	
SUPor-P-KG08841	28A	P{y[+mDint2] w[BR.E.BR]=SUPor-P}KG0884	P{SUPor-P}	FBti0024240	[24]	14972	
SUPor-P-KG09078	20D	P{y[+mDint2] w[BR.E.BR]=SUPor-P}KG09078	P{SUPor-P}	FBti0024820	[24]	14781	
SUPor-P-KG10047	60F 	P{y[+mDint2] w[BR.E.BR]=SUPor-P}KG10047	P{SUPor-P}	FBti0074117	[31]	-	
SUPor-P-KG10155	91F 	P{y[+mDint2] w[BR.E.BR]=SUPor-P}cdi[KG10155]	P{SUPor-P}	FBti0039088	[24]	16513	
P-w-y-T2R-PAR	60F 	P{y[+mDint2] w[BR.E.BR]=SUPor-P}T2R-PAR	P{SUPor-P}	-	Fig. S1	-	
T-1	50C 	Ab(2;3)T-1,P{w[+mC]=lacW}50C.x7/Cy	P{lacW}	FBti0027547	[1]	-	
wg-lacZ	27F 	P{ry[+t7.2]=PZ}wg[02657]/CyO	P{PZ}	FBti0005160	[20]	11205	

Table S2: Genotype and references of transgene(s) insertions tested as TSE silencers or targets.
