# Supplementary figures and images for: Telomeric Trans-Silencing in Drosophila melanogaster: Tissue Specificity, Development and Functional Interactions between Non-Homologous Telomeres
Source: PLoS One. 2008 Sep 22;3(9):e3249. doi: 10.1371/journal.pone.0003249 (PMC2547894; doi:10.1371/journal.pone.0003249)

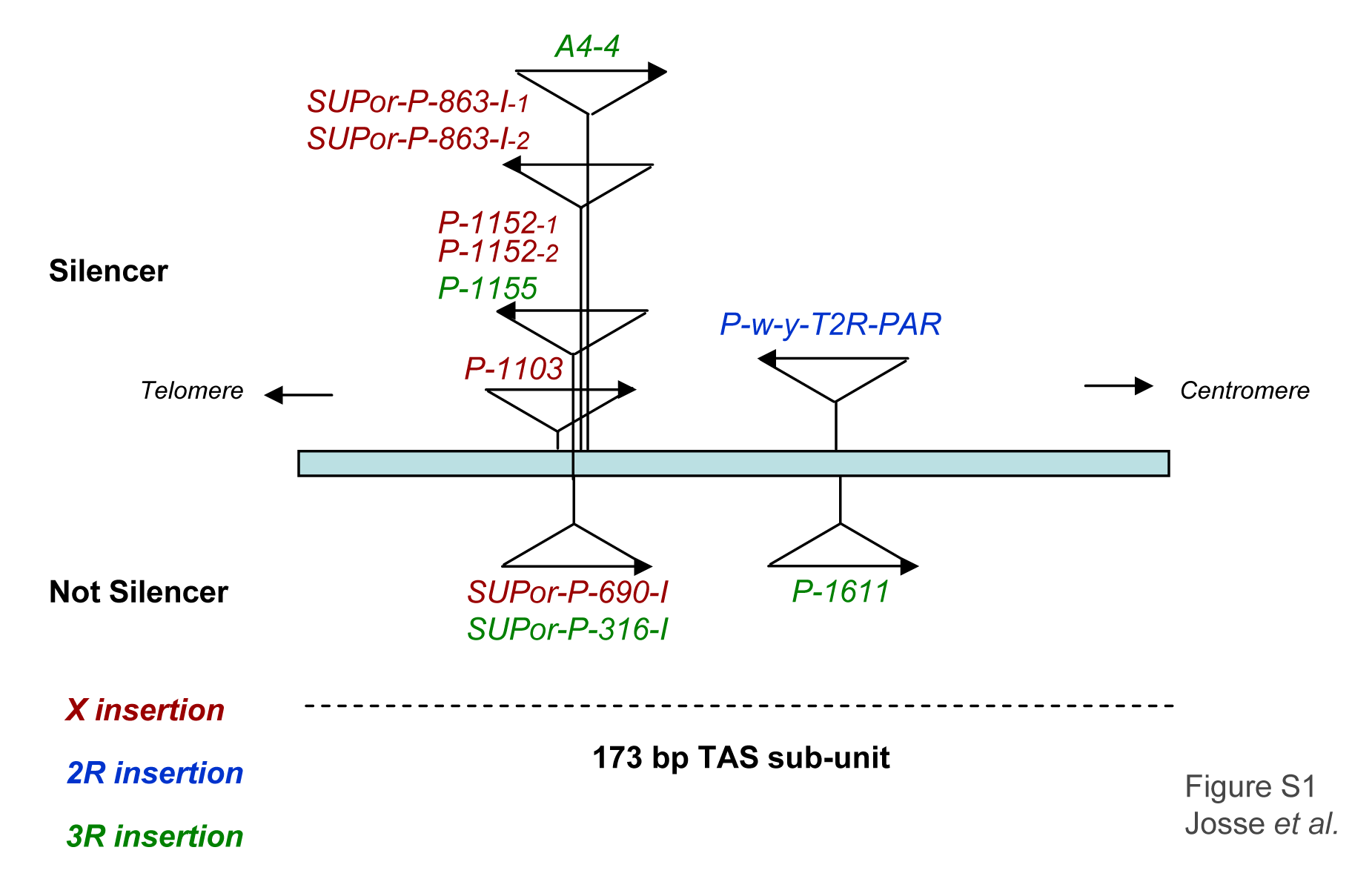

Supplement: Figure S1 — Position inside the TAS sequence of telomeric transgenes in relation to their repression capacities. All the transgenes analyzed here have been found to be localized inside a 173bp TAS subrepeat, a motif tandemly repeated (3–4 times) inside each TAS unit [1]. This 173bp motif was also found to be tandemly repeated in the TAS described for other telomeres (2R and 3R chromosomal arms) [2]. Transgenes therefore are drawn along a single 173bp subrepeat. For each transgene, the name, the orientation (arrowhead indicates the 3′ P element end) and the chromosomal arm (indicated by the color) is given. TSE silencer transgenes are positioned above the line whereas transgenes devoid of repression capacities are positioned below it. Both P-1152 and SUPor-P-863-I were found to carry two transgene copies at the same positions. The P-1152 copies are located at the same position in two adjacent TAS 173bp subrepeats and result likely from a 173bp subunit duplication. SUPor-P-863-I has a tandem repeat of two SUPor-P copies in direct orientation, this tandem being flanked by a target site duplication: this results likely from a transgene rearrangement which derived from a single initial transgene insertion. These copies are numbered 1 and 2 and are shown at the same position. A4-4 was described and mapped previously [2]–[6]. (See the additional file called “Figure Notes and References S1”). (0.38 MB TIF) [file pone.0003249.s003.tif]
