# Supplementary material for: Telomeric Trans-Silencing in Drosophila melanogaster: Tissue Specificity, Development and Functional Interactions between Non-Homologous Telomeres
Source: PLoS One. 2008 Sep 22;3(9):e3249. doi: 10.1371/journal.pone.0003249 (PMC2547894; doi:10.1371/journal.pone.0003249)
Supplement: Figure Notes and References S1 — Notes and references for Figure S1 (0.06 MB RTF) [file pone.0003249.s004.rtf]

Supporting Information.

Figure S1: Position inside the TAS sequence of telomeric transgenes in relation to their repression capacities.  
 	
 Introduction - Results
In an attempt to determine out why some telomeric transgenes inserted in TAS are TSE silencers, whereas others have no repression capacity, we investigated  the precise mapping and the sense of insertion for some of them within a TAS unit. These insertions  are located at different chromosomal arm telomeres (X, 2R, 3R).  This comparison was performed for different types of constructs. P-1152, P-1103, P-1155  and P-1611 are P-lacZ-rosy transgenes whereas  SUPor-P-863-I , SUPor-P-316-I, SUPor-P-690-I  and P-w-y-T2R-PAR  are P-white-yellow transgenes. The P-w-y-T2R-PAR line  required additional analysis. We received this stock with indications that it carried one SUPor-P  insertion located at the 2R chromosomal arm telomere; our mapping of the transgene by PCR analysis confirmed that it corresponded to an insertion in the TAS of the 2R telomere, but raised a doubt concerning the number of  the SUPor-P stock. We thus decided to rename the stock in our lab "P-w-y-T2R-PAR"  for  P-w-y-Telomere 2R-Paris. We confirmed that the "P-w-y-T2R-PAR"  transgene is located at the 2R telomere (60F) by in situ hybridization with a white probe. We also verified the structure of the transgene, thereby confirming the presence of the yellow and white markers (genetically). The precise mapping of the insertion site of "P-w-y-T2R-PAR" indicates that this insertion likely corresponds to the SUPor-P-KG08867, which is no longer available. A4-4 (also called P-833) is a P-white-rosy transgene which was described and mapped previously [2-6]. 


The TAS located at the X, 2R and 3R  telomeres share a well conserved tandem repeat (3-4 times) of a 173bp motif [1,2], identified as  deriving from a 5'LTR of the retrotransposon called  invader [2]  surrounded by a distal region and a proximal region which are less conserved between telomeres. These TAS units have been aligned and compared in the Supplemented data of  [2]   (http://www.nature.com/nature/journal/v450/n7167/abs/nature06263.html ). 
All the transgenes under investigation were mapped within a 173bp subrepeat and the schematic representation on Figure S1, indicating their relative positions, therefore utilizes a single 173bp subrepeat.
These data show that: 1- P-transgenes do not insert at random within TAS since the 173bp subrepeat is a hotspot and within this subrepeat, a number of transgenes are inserted at the same nucleotidic site. For example P-1152, P-1155, SUPor-P-690-I, and  SUPor-P-316-I have the same nucleotidic location even if  they are located at different telomeres or have different structures. This non random insertion of telomeric transgenes is consistent with previous studies [1,7]; 2- a silencer can be found in both orientations even if silencer transgenes are more frequently found in the 3'-5' orientation for P sequences relative to TAS. However, more observations are required to test if this preference is significant. 

Figure S1 - Materials and Methods 
Transgenes have been mapped using direct PCR amplification using primers specific of TAS sequences and primers specific of P transgenes and the presence of the Target Site Duplication was checked in each case.
PCR products were obtained for both extremities, cloned and sequenced, in order to find the target site duplication, indicative of a unique element (detailed protocol will be provided upon request).
Primers specific for P transgenes used for localizing the telomeric transgenes 
P89 				CGTCCGCACACAACCTTTCC
P2751 				CCACGGACATGCTAAGGGTTAA
LacZ877-900 			CGTAATGGGATAGGTTACGTTGG
Primers specific for TAS sequences used for localizing the telomeric transgenes
5046				CCTAGAATGCGAACAGTCACG
6820				GCCTCTGCAGCGATCGGGTT
TAS344-366			ACTATTCTTGACAACCCGATCGG
TAS509-486			AGGGAGATGAAAATGTAGTGAACG

Figure S1 - References
1. Karpen GH, Spradling AC (1992) Analysis of subtelomeric heterochromatin in the Drosophila minichromosome Dp1187 by single P element insertional mutagenesis. Genetics 132: 737-753.
2. Yin H, Lin H (2007) An epigenetic activation role of Piwi and a Piwi-associated piRNA in Drosophila melanogaster. Nature 450: 304-308.
3. Levis R, Hazelrigg T, Rubin GM (1985) Effects of genomic position on the expression of transduced copies of the white gene of Drosophila. Science 229: 558-561.
4. Levis RW (1989) Viable deletions of a telomere from a Drosophila chromosome. Cell 58: 791-801.
5. Levis RW, Ganesan R, Houtchens K, Tolar LA, Sheen FM (1993) Transposons in place of telomeric repeats at a Drosophila telomere. Cell 75: 1083-1093.
6. Levis R Personal Communication.
7. Biessmann H, Prasad S, Semeshin VF, Andreyeva EN, Nguyen Q, et al. (2005) Two distinct domains in Drosophila melanogaster telomeres. Genetics 171: 1767-1777.
