# Supplementary material for: Telomeric Trans-Silencing in Drosophila melanogaster: Tissue Specificity, Development and Functional Interactions between Non-Homologous Telomeres
Source: PLoS One. 2008 Sep 22;3(9):e3249. doi: 10.1371/journal.pone.0003249 (PMC2547894; doi:10.1371/journal.pone.0003249)
Supplement: Table References S2 — References for Table S2 (0.05 MB RTF) [file pone.0003249.s005.doc]

Supporting Information.

Table S2 references: 

1.	D R Dorer et S Henikoff, “Expansions of transgene repeats cause heterochromatin formation and gene silencing in Drosophila,” Cell 77, no. 7 (1994): 993-1002, doi:8020105. 
2.	T Hazelrigg, R Levis, et G M Rubin, “Transformation of white locus DNA in drosophila: dosage compensation, zeste interaction, and position effects,” Cell 36, no. 2 (1984): 469-81, doi:6319027. 
3.	B Lemaitre, S Ronsseray, et D Coen, “Maternal repression of the P element promoter in the germline of Drosophila melanogaster: a model for the P cytotype,” Genetics 135, no. 1 (1993): 149-60, doi:8224815. 
4.	D Higuet, D Anxolabéhère, et D Nouaud, “A particular P-element insertion is correlated to the P-induced hybrid dysgenesis repression in Drosophila melanogaster,” Genetical Research 60, no. 1 (1992): 15-24, doi:1333434. 
5.	H Lin et A C Spradling, “A novel group of pumilio mutations affects the asymmetric division of germline stem cells in the Drosophila ovary,” Development (Cambridge, England) 124, no. 12 (1997): 2463-76, doi:9199372. 
6.	K D Swanson et R Ganguly, “Characterization of a Drosophila melanogaster gene similar to the mammalian genes encoding the tyrosine/tryptophan hydroxylase activator and protein kinase C inhibitor proteins,” Gene 113, no. 2 (1992): 183-90, doi:1349290. 
7.	P Zhang et A C Spradling, “The Drosophila salivary gland chromocenter contains highly polytenized subdomains of mitotic heterochromatin,” Genetics 139, no. 2 (1995): 659-70, doi:7713423. 
8.	S Ronsseray, A Boivin, et D Anxolabéhère, “P-Element repression in Drosophila melanogaster by variegating clusters of P-lacZ-white transgenes,” Genetics 159, no. 4 (2001): 1631-42, doi:11779802. 
9.	P Deák et coll., “P-element insertion alleles of essential genes on the third chromosome of Drosophila melanogaster: correlation of physical and cytogenetic maps in chromosomal region 86E-87F,” Genetics 147, no. 4 (1997): 1697-722, doi:9409831. 
10.	W J Brook et coll., “Gene expression during imaginal disc regeneration detected using enhancer-sensitive P-elements,” Development (Cambridge, England) 117, no. 4 (1993): 1287-97, doi:8404531. 
11.	M Mlodzik et coll., “The Drosophila seven-up gene, a member of the steroid receptor gene superfamily, controls photoreceptor cell fates,” Cell 60, no. 2 (1990): 211-24, doi:2105166. 
12.	E Dequier et coll., “Top-DER- and Dpp-dependent requirements for the Drosophila fos/kayak gene in follicular epithelium morphogenesis,” Mechanisms of Development 106, no. 1-2 (2001): 47-60, doi:11472834. 
13.	R G Phillips et coll., “The Drosophila segment polarity gene patched is involved in a position-signalling mechanism in imaginal discs,” Development (Cambridge, England) 110, no. 1 (1990): 105-14, doi:2081453.
14.	T Lepage et coll., “Signal transduction by cAMP-dependent protein kinase A in Drosophila limb patterning,” Nature 373, no. 6516 (1995): 711-5, doi:7854456.
15.	U Heberlein, T Wolff, et G M Rubin, “The TGF beta homolog dpp and the segment polarity gene hedgehog are required for propagation of a morphogenetic wave in the Drosophila retina,” Cell 75, no. 5 (1993): 913-26, doi:8252627. 
16.	H J Bellen et coll., “P-element-mediated enhancer detection: a versatile method to study development in Drosophila,” Genes & Development 3, no. 9 (1989): 1288-300, doi:2558050. 
17.	P D Harvie, M Filippova, et P J Bryant, “Genes expressed in the ring gland, the endocrine organ of Drosophila melanogaster,” Genetics 149, no. 1 (1998): 217-31, doi:9584098. 
18.	S Roth et coll., “cornichon and the EGF receptor signaling process are necessary for both anterior-posterior and dorsal-ventral pattern formation in Drosophila,” Cell 81, no. 6 (1995): 967-78, doi:7540118. 
19.	E Martín-Blanco et coll., “puckered encodes a phosphatase that mediates a feedback loop regulating JNK activity during dorsal closure in Drosophila,” Genes & Development 12, no. 4 (1998): 557-70, doi:9472024. 
20.	A C Spradling et coll., “The Berkeley Drosophila Genome Project gene disruption project: Single P-element insertions mutating 25% of vital Drosophila genes,” Genetics 153, no. 1 (1999): 135-77, doi:10471706. 
21.	M Meise et W Janning, “Localization of thoracic imaginal-disc precursor cells in the early embryo of Drosophila melanogaster,” Mechanisms of Development 48, no. 2 (1994): 109-17, doi:7873401. 
22.	D H Castrillon et coll., “Toward a molecular genetic analysis of spermatogenesis in Drosophila melanogaster: characterization of male-sterile mutants generated by single P element mutagenesis,” Genetics 135, no. 2 (1993): 489-505, doi:8244010. 
23.	Annette Peter et coll., “Mapping and identification of essential gene functions on the X chromosome of Drosophila,” EMBO Reports 3, no. 1 (2002): 34-8, doi:11751581. 
24.	Hugo J Bellen et coll., “The BDGP gene disruption project: single transposon insertions associated with 40% of Drosophila genes,” Genetics 167, no. 2 (2004): 761-81, doi:15238527. 
25.	S Eaton et T B Kornberg, “Repression of ci-D in posterior compartments of Drosophila by engrailed,” Genes & Development 4, no. 6 (1990): 1068-77, doi:2384212. 
26.	Nicoletta Corradini et coll., “FISH analysis of Drosophila melanogaster heterochromatin using BACs and P elements,” Chromosoma 112, no. 1 (2003): 26-37, doi:12827380. 
27.	K Howard et coll., “Migration of Drosophila germ cells: analysis using enhancer trap lines,” Development (Cambridge, England). Supplement (1993): 213-8, doi:8049476. 
28.	L Marin et coll., “P-Element repression in Drosophila melanogaster by a naturally occurring defective telomeric P copy,” Genetics 155, no. 4 (2000): 1841-54, doi:10924479. 
29.	Anuranjan Anand et coll., “Drosophila " enhancer-trap" transposants: Gene expression in chemosensory and motor pathways and identification of mutants affected in smell and taste ability,” Journal of Genetics 69, no. 3 (1990): 151-68. 
30.	Pamela Geyer Personnal communication
31.	Harald Biessmann et coll., “Two distinct domains in Drosophila melanogaster telomeres,” Genetics 171, no. 4 (2005): 1767-77, doi:genetics.105.048827. 
32.	R R Roseman et coll., “A P element containing suppressor of hairy-wing binding regions has novel properties for mutagenesis in Drosophila melanogaster,” Genetics 141, no. 3 (1995): 1061-74, doi:8582613.
33.	E Bier et coll., “Searching for pattern and mutation in the Drosophila genome with a P-lacZ vector,” Genes & Development 3, no. 9 (1989): 1273-87, doi:2558049. 
34.	S Kobayashi et coll., “Two types of pole cells are present in the Drosophila embryo, one with and one without splicing activity for the third P-element intron,” Development (Cambridge, England) 117, no. 3 (1993): 885-93, doi:8391978.
35.	T Lukacsovich et coll., “Dual-tagging gene trap of novel genes in Drosophila melanogaster,” Genetics 157, no. 2 (2001): 727-42, doi:11156992.
36.	C J O'Kane et W J Gehring, “Detection in situ of genomic regulatory elements in Drosophila,” Proceedings of the National Academy of Sciences of the United States of America 84, no. 24 (1987): 9123-7, doi:2827169.
37.	R R Roseman, V Pirrotta, et P K Geyer, “The su(Hw) protein insulates expression of the Drosophila melanogaster white gene from chromosomal position-effects,” The EMBO Journal 12, no. 2 (1993): 435-42, doi:8382607.
38.	G M Rubin et A C Spradling, “Vectors for P element-mediated gene transfer in Drosophila,” Nucleic Acids Research 11, no. 18 (1983): 6341-51, doi:6312420.
39.	C Wilson et coll., “P-element-mediated enhancer detection: an efficient method for isolating and characterizing developmentally regulated genes in Drosophila,” Genes & Development 3, no. 9 (1989): 1301-13, doi:2558051. 
